# Topological augmentation to infer hidden processes in biological systems

**DOI:** 10.1093/bioinformatics/btt638

**Published:** 2013-12-02

**Authors:** Mikael Sunnåker, Elias Zamora-Sillero, Adrián López García de Lomana, Florian Rudroff, Uwe Sauer, Joerg Stelling, Andreas Wagner

**Affiliations:** ^1^Department of Biosystems Science and Engineering/Swiss Institute of Bioinformatics, ETH Zurich, 4058 Basel, Switzerland, ^2^Competence Center for Systems Physiology and Metabolic Diseases, ETH Zurich, 8093 Zurich, Switzerland, ^3^Institute of Evolutionary Biology and Environmental Studies/Swiss Institute of Bioinformatics, University of Zurich, 8057 Zurich, Switzerland, ^4^Institute for Molecular Systems Biology, 8093 Zurich, Switzerland and ^5^The Santa Fe Institute, Santa Fe, 87501 New Mexico, USA

## Abstract

**Motivation:** A common problem in understanding a biochemical system is to infer its correct structure or topology. This topology consists of all relevant state variables—usually molecules and their interactions. Here we present a method called topological augmentation to infer this structure in a statistically rigorous and systematic way from prior knowledge and experimental data.

**Results:** Topological augmentation starts from a simple model that is unable to explain the experimental data and augments its topology by adding new terms that capture the experimental behavior. This process is guided by representing the uncertainty in the model topology through stochastic differential equations whose trajectories contain information about missing model parts. We first apply this semiautomatic procedure to a pharmacokinetic model. This example illustrates that a global sampling of the parameter space is critical for inferring a correct model structure. We also use our method to improve our understanding of glutamine transport in yeast. This analysis shows that transport dynamics is determined by glutamine permeases with two different kinds of kinetics. Topological augmentation can not only be applied to biochemical systems, but also to any system that can be described by ordinary differential equations.

**Availability and implementation:** Matlab code and examples are available at: http://www.csb.ethz.ch/tools/index.

**Contact:** mikael.sunnaker@bsse.ethz.ch; andreas.wagner@ieu.uzh.ch

**Supplementary information:** Supplementary data are available at *Bioinformatics* online.

## 1 INTRODUCTION

Cellular processes, for instance in metabolism, signaling or transport, can often be modeled by sets of deterministic differential equations that describe concentration changes in the molecular species of interest over time. However, the kind of molecular interactions and the specific biochemical form they take in a cellular process are frequently uncertain. Such topological or *structural* uncertainty has been previously tackled by defining a set of candidate models (e.g. see Kuepfer *et al.*, 2007; Toni and Stumpf, 2010; Xu *et al.*, 2010) that reflects different mechanistic hypotheses. Each of these candidates could in principle encapsulate the process, and empirical data can be used to discriminate between them using available methods for statistical inference (Akaike, 1973; Kass and Raftery, 1995).

A severe limitation of evaluating all candidate models is that their number grows exponentially with the number of uncertainties in the model topology. To preselect a subset of candidate models that is small enough to be analyzed, one can incorporate all hypotheses into a single *master* model, which is then reduced by elimination of hypotheses (Floettmann *et al.*, 2008; Sunnåker *et al.*, 2013b). However, the resulting subset of models may not contain any model that satisfactorily explains the experimental data. Furthermore, the number of model parameters increases with the number of hypothetical mechanisms, and model reduction may become infeasible due to the ‘curse of dimensionality’ (Sunnåker *et al.*, 2013a). If no satisfactory model emerges from model reduction, or if the number of hypothetical mechanisms is too large, it may be best to start the inference process from a smaller model that is successively extended and improved. However, there are few computational methods available to extend a model by systematically identifying missing terms in a differential equation, or by improving the existing terms.

Kristensen *et al.* (2005) have suggested using stochastic differential equations (SDEs; Øksendal, 2003) instead of ordinary differential equations (ODEs) for model construction. In addition to deterministic terms as in ODEs, SDEs comprise stochastic terms that account for uncertainty in the realization of trajectories, and the equations’ solution takes the form of a probability distribution. The method by Kristensen *et al.* (2005) exploits that stochastic equation terms may fill the gap between the model predictions and the experimental data, and point to the deterministic part of a model’s equations that can be improved. This is because the estimated level of uncertainty in the prediction of state variables dictates the impact of each data point on the estimated model response. Model improvements result in a reduction of the estimated magnitude of the stochastic terms, and the remaining stochastic terms can then be used to pinpoint model deficiencies. We note that the incorporation of non-measured variables is commonly used in (linear and discrete-time) dynamic Bayesian network models used to study genetic regulatory networks (Beal *et al.*, 2005; Perrin *et al.*, 2003; Wu *et al.*, 2004). However, the aim of these methods is rather to compensate for unknown regulators than to infer unmodeled parts explicitly. Other approaches based on a combined space of parameters and model structures are computationally expensive due to a combinatorial explosion in model terms (Nachman *et al.*, 2004; Schmidt *et al.*, 2011).

Here, we propose a novel computational method for model inference. We call it *topological augmentation*. It is based on the ideas by Kristensen *et al.* (2005) on how to separate uncertainty in model predictions from measurement noise. In contrast to previous approaches, it bases conclusions about the system on characterization of and integration over the parameter space. The conclusions are not biased by a single parameter point, but they are valid over a biologically meaningful range of parameter values. The approach also naturally connects to Bayesian methods for model inference, where the probability of different hypotheses is based on prior knowledge, and can be iteratively updated with experimental observations.

## 2 METHODS

### 2.1 Topological augmentation

We consider deterministic models in the form of systems of ODEs, where the state variables describe the concentrations of molecules. Such a model, which we refer to as 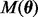, takes the form as follows:
(1)
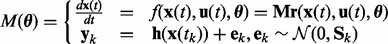

with the state variables 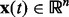 (i.e. there are *n* state variables; other variable numbers are similarly defined), the potentially time-varying inputs 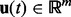 and the vector of model parameters 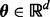. The function 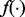 (where the dot abbreviates the function arguments) is, in general, a non-linear vector field that describes the dynamics of the state variables, and may also be expressed as the stoichiometric matrix 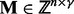 (integer entries) times the reaction vector 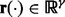. The model output 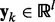 at time point *t_k_*, 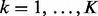 is generated by a non-linear function 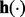 of the system state variables 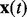 and an additive contribution of measurement noise 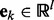. Furthermore, the measurement noise is normally distributed with covariance matrix 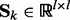. The available experimental data are denoted by 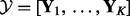, where the subscript denotes the observation time point. Topological augmentation aims at inferring the form of 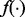, given a (biological) system, from a set of experimental data. Note that 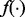 represents the form of the interactions (e.g. chemical reactions) between the state variables. Correctly modeled interactions are characterized by a small difference between model predictions and experimental data, and this difference is reduced through successive improvements of 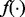.

**Fig. 1.**
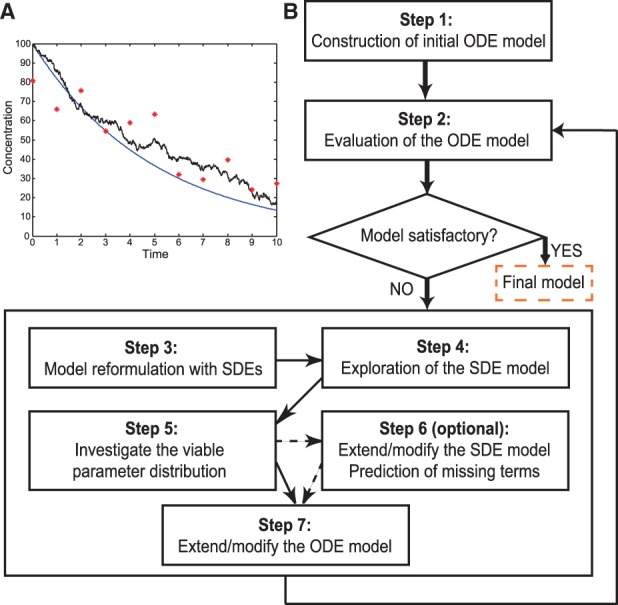
Flow chart for the computational method. (**A**) Simulation of a linear ODE model with one compartment of the form: 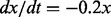 (smooth trajectory), as well as the corresponding SDE model: 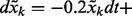 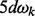, 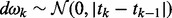 (fluctuating trajectory). In both models the initial condition 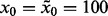, the output 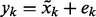 and 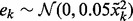 (time index: 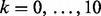). Artificial data *y_k_* are denoted by stars. (**B**) The method, and model inference process, starts from a basic model with a minimal set of mechanisms. If the basic model is not sufficient, the ODEs are reformulated as SDEs, and the SDE model is explored. The distribution of viable parameter points and (optionally) an extended SDE model for detailed predictions are used to guide improvements of the ODE model. The procedure is repeated for each generated model until a sufficiently descriptive model has been constructed

Models can contain two main sources of uncertainty, commonly referred to as system noise and measurement noise. System noise can be further decomposed into two parts, intrinsic noise and topological uncertainty. Intrinsic noise stems from non-determinism (e.g. random effects due to small numbers of particles). Topological uncertainty reflects an incomplete understanding of system components and their interactions, which leads to model components that are poorly specified or missing. This uncertainty results in model predictions that conflict with experimental observations, e.g. when an indispensable negative feedback in an oscillating signaling pathway is unknown to the modeler. System noise can then be incorporated into model predictions, to represent all processes that are not explicitly described by the ODE model, by formulation of an SDE model. Figure 1A illustrates the different noise sources revealed by simulation of an SDE model. The ODE solution (smooth trajectory), which describes the concentration of a hypothetical molecule, may after incorporation of system noise take the form of the SDE solution (fluctuating trajectory) for a particular realization. The asterisks indicate the other major source of noise in the data, i.e. measurement noise, which we added to the SDE model solution to generate *in silico* a response corresponding to experimental measurements.

To infer system properties, it is important that we can separate the signal (information) from noise, but to identify targets for model improvements the system noise must also be separable from measurement noise. Assume that both types of noise are present at each measurement time point. The optimal estimate of the molecule concentration in the system then neither coincides with the average model predictions (due to system noise) nor with the average experimental measurements (due to measurement noise). To compute an accurate estimate, we therefore need to balance the incorporation of information from the model predictions to the information from the experimental data. If parts of the system are unmodeled, the concentration of the molecule is better estimated by incorporation of system noise. The influence of each experimental data point on the estimates is then stronger than without the system noise (see Supplementary Data, section 2). If the system noise does not stem from intrinsic noise, we can infer that the ODE model is missing a part, which leads to incorrect estimation of the molecule’s concentration.

Topological augmentation systematically quantifies and reduces topological uncertainty by identifying correct deterministic system components in an iterative fashion (Fig. 1B). In *step 1*, we construct an initial ODE model based on well-known core components of the studied system; no hypothetical mechanisms are included in the model at this stage. Typically, elementary reactions derived from basic kinetic principles, such as mass action kinetics, will be incorporated in such an initial ODE model.

In *step 2*, we evaluate the ODE model defined previously by investigating whether the model is compatible with the available experimental data. We have previously defined a formal viability criterion for parameter points in ODE models based on the expected log-likelihood for a model with a parameterization that captures all regularities in the data:
(2)
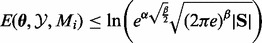

where parameter point 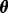 is viable in model *M_i_* if Equation (2) is satisfied, α is the acceptable deviation in number of standard deviations for parameter viability, 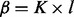 is the number of data points and 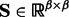 is a diagonal covariance matrix for the measurement noise with the block matrices 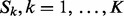 in the diagonal [see Sunnåker *et al.* (2013b) and Supplementary Data, section 3]. Aspects of the model predictions not captured by the viability criterion should also be checked, e.g. that the model predictions do not systematically over- or underestimate the observations. We characterize the part of a model’s parameter space that is compatible with experimental data instead of assessing model quality at a single (optimal) parameter point. This is particularly important if the model parameter values are not uniquely identifiable because different parameter points may render different predictions of unobserved variables. If the model fit is satisfactory, there is no reason to further improve the model structure until new incompatible observations have been obtained.

*Step 3* involves reformulation of the ODE model from step 1 [Equation (1)] as a system of SDEs:
(3)
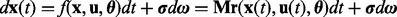

where 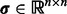 quantifies the uncertainty in the model predictions and 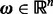 is a Wiener process, a time-continuous stochastic process whose variance increases linearly with time (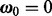 and 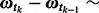 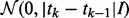). The SDEs are written in differential form, as 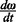 cannot be treated analytically (Overgaard *et al.*, 2005). The model in Equation (3) reduces to the form of the ODE models in Equation (1) if 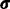 vanishes. The additional term 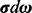 represents the system noise, i.e. the combined effect of inherent noise and topological uncertainty. The system noise can only be completely eliminated through model improvements if the inherent noise is assumed to be negligible (i.e. for a large number of molecules). The system noise term of an SDE is commonly referred to as the diffusion term, whereas the deterministic term 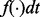 is referred to as the drift term. If the coefficients of the Wiener process (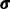) cannot be experimentally measured, they can instead be parameterized and estimated from the experimental data.

*Step 4* explores the parameter space of the SDE model. The SDEs in Equation (3) comprise three types of tunable parameters: ODE model parameters 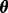, elements of the matrix 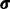 and elements of the set of measurement covariance matrices 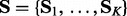, and we denote the set of all potential parameters by 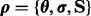. Parameter points of ODE models are typically evaluated based on objective functions that rely on the (least squares) distance between model predictions and experimental data. However, the elements of 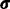 and **S** cannot be estimated by comparing model simulations to data, as the generated state trajectories are different for each simulation of the SDE model. Following Overgaard *et al.* (2005), we surmount this issue with an extended Kalman filter modified for SDEs (Kristensen *et al.*, 2005) (see Supplementary Data, section 2). Based on the entries of **S** and 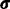, the Kalman filter assigns ‘weights of trust’ to the experimental data and to the simulations at the experimental time points, separating noise from signal in the experimental data. For negligible values of **S** (and non-negligible values of 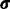), the Kalman filter predictions coincide with the experimental measurements. On the other hand, for negligible values of 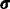 (and non-negligible values of **S**) the Kalman filter’s predictions equal the ODE model predictions. Therefore, we can estimate **S** and 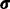 by varying the corresponding parameters.

The quality of a model with a certain parameterization is measured by a cost function 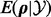 [see Supplementary Data and Equation (5)]. The evaluation of a given parameter point with the Kalman filter maps to a unique value of the cost function (despite the use of SDEs). Each evaluated parameter point is classified as viable or non-viable, for a given cost function cutoff value, and we refer to the union of the regions of viable parameter points as the viable space. Because the viability criterion for ODE models [Equation (2)] is not valid for SDE models, we instead define a viability cutoff based on the distance to the optimal parameter point (for details see Supplementary Data, section 3). We then use the method by Zamora-Sillero *et al.*, 2011 to sample the parameter space and to characterize the viable space. In the first part of this method, the parameter space is sampled as broadly as possible with a variant of the Metropolis–Hastings Markov chain Monte Carlo method that is designed for this purpose. The high-likelihood regions identified in the first step are then characterized in detail in the second step, with an approach based on ellipsoid expansions [see Supplementary Data, section 4 and Zamora-Sillero *et al.*, 2011 for details].

In *step 5*, we investigate the viable parameter distribution to identify model parts with potential for improvements. Small entries of 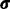 in the viable region of parameter space indicate that 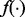 correctly represents the underlying system. In contrast, large entries of 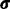 indicate room for model improvements. Because each entry of 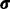 corresponds to one specific SDE in the model, one can pinpoint specific equations that are sensible candidates for modifications. We identify non-negligible entries of 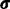 by visual inspection, but heuristics may be used to automatize the process.

In *step 6*, we extend the SDE model based on the analysis in step 5 to predict missing model parts in step 7. This step is optional, intended to provide additional support for the decision process in step 7. The SDE model extensions should be incorporated as additional state variables in 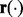, or by extending 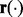 with additional reactions for entries of 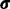 that are non-negligible. For the additional state variables, 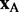, we define the following SDEs:
(4)
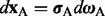

where the trajectories of the state variables 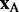 are determined solely by the diffusion terms. In models for which there are reasons to suspect that a particular parameter is not constant, it may be useful to reformulate that parameter into a state variable [together with an SDE in the form of Equation (4)]. After exploring the parameter space of the extended SDE model, the SDE’s behavior is simulated for viable parameter points to infer the trajectories of the additional state variables 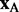. The Kalman filter used for parameter space explorations can also be used to infer the trajectories of 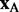 in time because 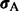 and the drift term of the SDE model from step 3 together determine how the state variables evolve. The additional terms 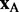 may improve the SDE model by compensating for missing model parts (despite the unknown mathematical form of the drift term).

In *step 7*, we make an informed decision about reaction terms to be added to the ODE model based on the information from step 5 (reactions to which these terms should be added) and step 6 (form of the reaction terms). In accordance with Occam’s razor, we add reaction terms that are as simple as possible. For example, the term 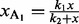, where *k*_1_ and *k*_2_ are parameters, is reasonable if 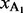 becomes saturated over time (where the increasing state variable 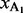 is a function of *x*).

After step 7, we return to step 2 to evaluate the extended ODE model. If this model is satisfactory, the model construction process is complete, if not, steps 3–7 are repeated. Once a satisfactory model has been constructed it can be useful to compare the performance of the ODE models (and potentially SDE models) that were generated in the process. To compare two models *M_i_* and *M_j_*, given experimental data 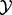 and known measurement noise **S**, we compute the Bayes factor (Kass and Raftery, 1995):
(5)
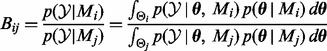

where 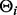 and 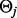 are the parameter spaces for models *M_i_* and *M_j_*, respectively. Bayes factors estimate the relative plausibility of two models, and they directly relate to the ratio of posterior model probabilities by:
(6)
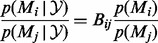

For equal prior model probabilities, 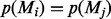, the Bayes factor equals the ratio of the posterior model probabilities.

### 2.2 Pharmacokinetic model

To illustrate the workflow of topological augmentation, we reinvestigate a pharmacokinetic model for the absorption of an orally administered drug into the bloodstream (Kristensen *et al.*, 2005). The model has two state variables that represent the availability of the drug in the gastrointestinal tract (*Q*, mg) and in the blood plasma (*C*, mg/l):

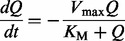

(7)
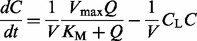

The model’s four parameters are 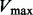 (maximal transport rate from the gastrointestinal tract to the blood plasma), 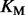 (the concentration of Q that gives a half-maximal reaction rate), 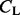 (clearance rate of the drug in the blood plasma) and *V* (volume of the blood plasma). We assume that *C* can be measured at *K* discrete time points, and we use a proportional measurement error model as Kristensen *et al.* (2005):
(8)
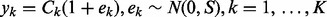

to generate an *in silico* set of 20 data points (for details see Supplementary Data, section 5).

**Fig. 2.**
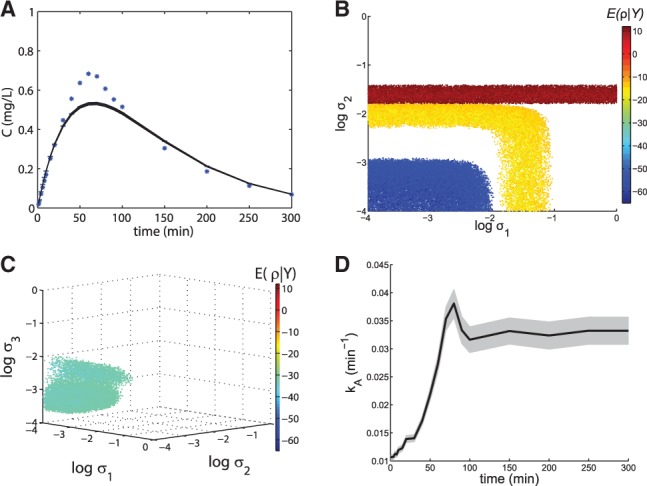
Pharmacokinetic model. (**A**) Fit of the linear pharmacokinetic model to the synthetic data (stars) for state variable C. To save computational time, the prediction was based on a randomly drawn subset (10 000 points) of all identified viable parameter points. (**B**) Projection of the six-dimensional parameter space onto the plane formed by parameters 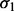 and 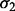 in the interval 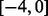 (log-space), for the linear model (yellow), final MM model (blue) and the MM model with 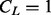 (red). The scale bar shows the negative log-likelihood of ρ given 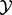 for the viable parameter points (indicated by the color). (**C**) Projections of the viable space into 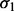, 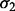 and 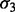 for the linear three state variables pharmacokinetic model (viability threshold: –31.5). (**D**) Predicted evolution of state variable 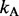 in the linear three state variables pharmacokinetic model. The shaded regions correspond to the (likelihood) weighted model predictions mean plus/minus one weighted standard error (Gatz and Smith *et al.*, 1995) of *C* (**A**) and *k_A_* (**D**), respectively

Let us now assume that the absorption kinetics described by the model’s non-linear term is unknown. A reasonable first representation of the drug kinetics is a linear uptake term modeled with mass action kinetics, in combination with a linear term for the degradation of the drug concentration in the blood plasma. However, this model cannot describe the observational data well: it tends to systematically overestimate or underestimate the synthetic data (see Fig. 2A).

The SDE model, in step 3, takes the form [Equation (3)] as follows:
(9)
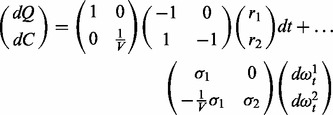

where the two reactions in the linear model are as follows:
(10)
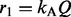

with parameter *k_A_*, and the internal drug degradation reaction:
(11)
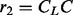

The initial linear ODE model corresponds to setting 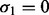 and 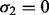. If the initial conditions and measurement noise *S* are known then five model parameters are unknown (*k_A_*, *C_L_*, *V*, 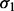 and 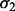).

We explored the parameter space of the SDE model over a broad range of values (eight orders of magnitude) for all five unknown parameters (step 4). The cost function Figure 2B (yellow region) shows the projections of the viable parameter points to 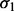 and 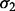. The viable parameter space is visualized in logarithmic space in all figures.

In step 5, we investigate the organization of the viable space. Its projection onto the axes 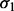 and 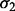 has roughly the shape of a boomerang (Fig. 2B). Importantly, there is no viable parameter point for which the values of both 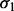 and 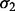 are negligible simultaneously. Kristensen *et al.* (2005), who analyzed the same model with a method based on a single parameter set, concluded that 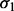 (but not 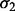) is necessary to explain the observational data. Topological augmentation shows that this assertion is correct only in some regions of the parameter space (the lower right part of the boomerang-shaped region) but incorrect in other regions (the upper left part). This illustrates the importance of characterizing a model’s behavior for a large set of parameters.

The distribution of viable parameter points indicates that the ODE model needs to be improved, but it is not immediately clear which of the two reactions should be targeted because inclusion of either 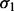 or 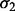 is sufficient to explain the data, depending on the region of the parameter space. Because only *C* is measured, 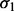 cannot be used to correct for misspecifications in *r*_2_; this would introduce an error in *r*_1_ through the first SDE. However, 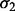, which only appears in the second SDE, can be used to correct *r*_1_ without introducing additional structural errors. To illustrate this idea, we fixed 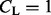 (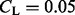 for the data). The viable parameter points projected onto 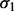 and 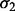 in Figure 2B (red region) indicate that 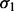, but not 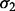, can be eliminated. Hence, reaction *r*_1_, as defined in Equation (10), should be improved. However, this can not be inferred directly from parameter optimizations, or explorations of the viable parameter space.

We attempt to determine the correct form of reaction *r*_1_ by creating an extended SDE model (step 6) where parameter 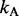 in *r*_1_ is considered as a state variable (Kristensen *et al.*, 2005):
(12)
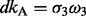

This reformulated SDE model defined by Equations (9) and (12) enables us to evaluate whether and how reaction *r*_1_ can be improved. The projections of the viable parameter space (Fig. 2C) show that it is necessary and sufficient to include 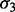: there are viable parameters close to the axes for 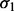 and 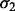, but not for 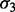. Hence, if we can find the correct form of 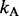, the corresponding ODE model will be compatible with the observational data. To determine the form of 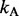, we use the extended Kalman filter to predict the trajectory of state variable 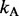. Although 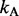 increases to saturation (Fig. 2D), the external drug concentration decreases until depletion in the same time interval (Supplementary Fig. S1). Therefore, a reasonable expression for 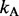 is as follows:
(13)
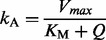

where *V_max_* and 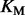 are additional model parameters.

In step 7, we use this expression for 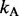 to construct a new SDE model on the form of Equation (9) but with 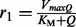 (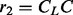). The projection of the model’s viable parameter points onto 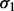 and 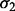 (Fig. 2B, blue region) shows that now both parameters are negligible, as viable points exist in the lower left corner of the parameter region. The posterior probabilities [Equations (5) and (6)] for the eight models (*M*_1_–*M*_8_) that can be constructed by eliminating combinations of 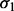 and 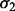 from the linear SDE model and from the non-linear SDE model (see Supplementary Table S3) indicate that the final non-linear ODE model *M*_8_ is >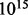 times more probable to be correct than the initial linear model *M*_4_. *M*_8_ has the form of Equation (7), which is also the model we used to generate the *in silico* data.

### 2.3 Glutamine transport in yeast

Environmental perturbations may provoke global changes in the regulation of a cell’s metabolome and transcriptome (Moxley *et al.*, 2009). In particular, yeast (*Saccharomyces cerevisiae*) cells respond to the availability of nitrogen sources in the environment with clear preferences. Nitrogen-rich sources such as glutamine or ammonium directly activate the so-called nitrogen catabolite repression (NCR) mechanism (via Gln3, Gat1, Dal80 and Gzf3), which is not activated for poor nitrogen sources such as proline or urea (Hofman-Bang, 1999). However, nitrogen-limited conditions trigger a response in the target-of-rapamycin pathway, which concomitantly activates the NCR-repressed genes via the Gln3 transcription factor (Georis *et al.*, 2009).

Four glutamine permeases are known in *S.cerevisiae*: Gap1 (Risinger *et al.*, 2006), Gnp1 (Zhu *et al.*, 1996), Agp1 (Schreve *et al.*, 1998) and Dip5 (Regenberg *et al.*, 1998). Transport can occur against a glutamine gradient due to an antiport mechanism that expels 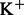 ions. Regulation and transport capabilities of the permeases are heterogeneous. Cells repress the expression of Gap1 and Agp1 under nitrogen-rich conditions, but not of Gnp1 or Dip5. Furthermore, permease affinities for glutamine are in the millimolar range for Gnp1, Agp1 and Dip5, but in the micromolar range for Gap1. Such complexity is required for the homeostasis of amino acids in the cell. The lack of regulation of the corresponding permeases leads to inhibition of cell growth and lethal cytotoxic effects due to amino acid imbalance (Risinger *et al.*, 2006).

**Fig. 3.**
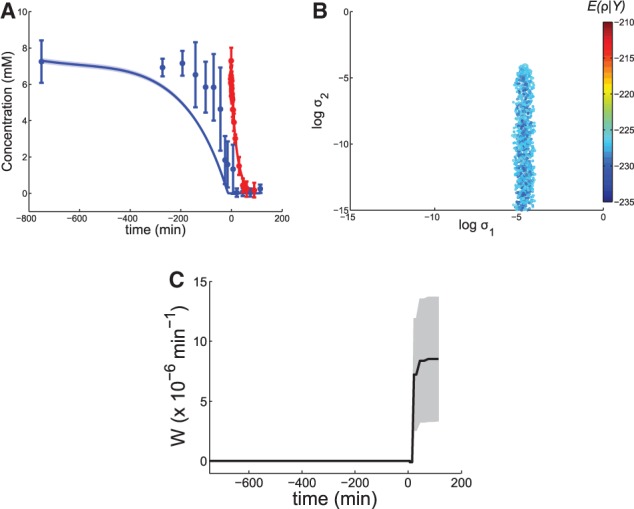
Yeast glutamine transport. (**A**) Experimentally determined intracellular (red circles) and extracellular (blue circles) glutamine concentrations and MM model trajectories (lines). The metabolic shift starts at time point 0 min. The weighted mean and the (small) weighted standard error (Gatz and Smith *et al.*, 1995) of the trajectories are shown. **(B**) Projection of the viable space for the SDE version of the MM model into 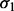 and 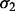 (parameter points whose likelihood is within five orders of magnitude from the most likely parameter point are considered viable). (**C**) Weighted mean prediction of state variable 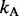 (black curve) for the extended SDE version of the MM model, where the gray area is the weighted standard error of the mean for the viable parameter points

To infer the relevance and roles of individual glutamine permeases during a metabolic shift, we grew a *S. cerevisiae* batch culture on a medium with both glutamine and proline as nitrogen sources (see Supplementary Data, section 6). During the initial steady state growth the culture consumes the preferred nitrogen source glutamine exclusively, and on glutamine depletion a metabolic shift to proline consumption occurs. We studied this dynamic transition by recording 14 and 23 data points for intracellular and extracellular glutamine concentrations, respectively (Fig. 3A).

The permeases’ functional diversity complicates the search for a correct dynamic model of glutamine transport. Therefore, we applied topological augmentation to infer a model of the yeast glutamine uptake process. In our starting simplistic transport model, all of the four permeases are functionally identical and the single glutamine uptake reaction is described by Michaelis–Menten (MM) kinetics (see also Supplementary Data, section 7.1):
(14)
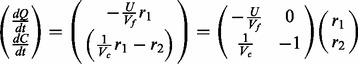

where 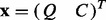 with *Q* and *C* the glutamine concentrations in the medium and in an average cell, respectively, *U* represents the number of cells at a given time (Supplementary Fig. S4) and *V_c_* and *V_f_* are the cellular and the culture medium volumes, respectively. The single glutamine uptake reaction has the rate 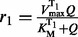, where 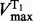 is the maximum rate of glutamine transport and 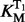 is the concentration of external glutamine for which the transport rate is half-maximal. The intracellular glutamine degradation reaction has the form 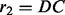, where *D* is the rate parameter for the degradation of *C* (Supplementary Data, section 7.1).

Although viable parameter points exist for this model (Supplementary Fig. S5), the dynamics of the intracellular glutamine concentration is systematically underestimated (Fig. 3A). Therefore, we reformulated the ODE model into an SDE model (see Supplementary Data, section 7.1).

The organization of this SDE model’s viable space (Fig. 3B) reveals that a non-negligible 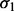 improves the model, whereas 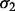 can be eliminated. The distribution of viable values for 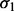 indicates an inconsistency between reaction *r*_1_ and the corresponding reaction in the system. Hence, the model may not yet capture the different functions of the permeases well. Gap1 is subject to tight dynamic regulation (Risinger *et al.*, 2006), which reinforces the idea of introducing an additional term in the MM model. Quantitative experimental data by Risinger *et al.* (2006) showed that the reversible activation of Gap1 is due to amino acid depletion. Therefore, we constructed an extended model version (step 6) with a new state variable *W*:
(15)
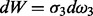

where 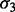 is a parameter. Reaction 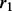 contains a new hypothetical term, 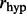, in a modified form:
(16)
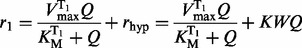



Initially, the variable 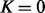 and 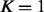 whenever 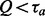, where parameter 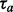 is inferred from experimental data (Supplementary Data, section 7.1). With this choice of *K*, we anticipated that the extra reaction term becomes important for small external glutamine concentrations. We then explored the viable parameter space for the extended SDE model and simulated the trajectories for *W* (Fig. 3C; see also Supplementary Fig. S6 illustrating that individual parameter points may be less predictive). Strikingly, the model predicts that the contribution of 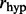 is negligible until *W* rapidly increases ∼15–20 min after the metabolic shift starts. It continues to increase gradually, but more slowly for around 100 min, which resembles a previously observed activation pattern for glutamine permeases under NCR control (Supplementary Fig. S7). Afterwards, *W* saturates and it remains constant until the end of the experiment. However, the SDE model is not the final result but a step in the process to infer a proper ODE model.

**Fig. 4.**
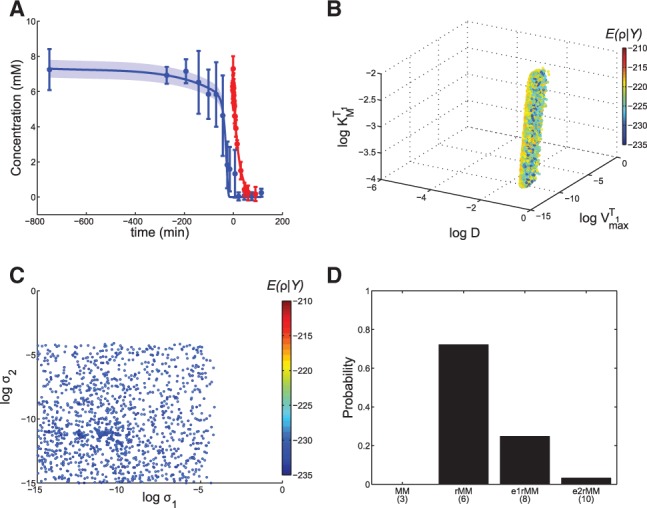
The rMM model results. (**A**) Experimental data for external (blue dots) and intracellular (red dots) glutamine concentrations and model simulations [weighted mean (solid lines) and weighted standard error (Gatz and Smith *et al.*, 1995) (shaded regions) of the predicted trajectories constructed from the uniformly sampled viable space]. (**B**) The six-dimensional viable space projected into three structural parameters. We uniformly sampled the region that contains viable parameter points. The cost function value associated with each parameter point, 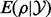 [Supplementary Data, Equation (5)], is mapped onto a color scale. (**C**) Projection of viable points (within five orders of magnitude from the most likely parameter point) to 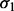 and 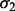 for the SDE version of the rMM model. (**D**) Posterior probabilities for glutamine transport models (number of parameters in parentheses; for convergences see Supplementary Fig. S11)

Next, we constructed an extended model of cellular glutamine uptake (step 7). We used the predictions of the extended SDE model to construct a regulated MM (rMM) model consisting of two MM terms that account for two independent transport regimes, with and without NCR control (see Supplementary Data, section 7.2). Gnp1 and Dip5, which are not subject to NCR regulation, have millimolar affinities (Regenberg *et al.*, 1998; Zhu *et al.*, 1996). Of the regulated terms only Agp1 has an affinity in the millimolar range, but not Gap1, whose affinity is in the micromolar range. To discriminate between the roles of Agp1 and Gap1, we first investigated both glutamine affinity parameters of the rMM model in the millimolar range. The descriptive power of the rMM model is notably improved compared with the MM model (Fig. 4A).

To investigate whether the rMM model (with affinities in the millimolar range) could be further improved or not, we reformulated the ODE model as an SDE model. Its viable space, shown in Fig. 4C, suggests that 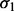 and 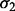 can be simultaneously eliminated, which means that the 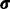 parameters cannot guide further modeling efforts. However, as yeast cells harbor four different glutamine permeases, we also investigated two extended ODE models that incorporate additional glutamine uptake reactions. Model e1rMM is based on the rMM model, but an additional MM term accounts for the activity of a third permease (in the millimolar range). This model accounts for the potentially different roles of Gnp1 and Dip5. Model e2rMM incorporates yet another regulated MM term (in the micromolar range corresponding to Gap1) to allow for different roles for all four permeases (see Supplementary Data, section 7.3 and Figs S9 and S10).

To compare the performance of all four candidate models, we computed the posterior probabilities for the models (see Fig. 4D). This let us conclude that the rMM model is the best model. It has two MM terms that correspond to two transport regimes, both operating in the millimolar range. They represent a constitutively active transport mechanism and the action of a permease that is specifically regulated for low external glutamine concentrations, respectively. A straightforward interpretation of these results is that the second transport mechanism is dominated by Agp1 rather than Gap1 under our experimental conditions. Additionally, the rMM model predicts that the activity of the second transporter is triggered by low levels of external glutamine, at ∼4 mM (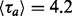 mM in Supplementary Fig. S8A). Finally, we conclude that topological augmentation helped us to infer these aspects of glutamine transport, and therefore is likely to prove useful for inference of various other aspects of biochemical systems.

## 3 DISCUSSION

Topological augmentation is a method designed to infer biochemical models in the face of uncertainties about their structure. It classifies and quantifies topological uncertainties that emerge from experimental observations. The method starts from an (usually too simple) ODE model, which it reformulates with SDEs, and relies on the distribution of viable parameter points obtained from random sampling to reveal the presence and the form of missing or incomplete reactions. Motivated by current gaps in the biological understanding of glutamine transport in yeast, we developed a model for glutamine transport and generated experimental data for topological augmentation. Interestingly, this analysis indicated a subsidiary role of Gap1 in the glutamine uptake process under the studied experimental conditions. In contrast to most other methods for model inference, we use observational data to explicitly guide the attention of the modeler to mechanisms for which there is room for improvements. Related, earlier SDE-based approaches evaluate an SDE model at a single parameter point (Kristensen *et al.*, 2005). Predictions based on a single parameter point can be misleading, as demonstrated by our pharmacokinetics application, even in combination with a local sensitivity analysis. Our extended approach that incorporates a distribution of viable parameter points provides substantially more information about potential model improvements. We showed that it can not only pinpoint missing reaction terms but also help finding the mathematical form of the missing terms.

We see five potential limitations of topological augmentation: first, sampling parameter spaces is computationally more costly than identifying a single optimal parameter point; this limitation could be overcome by future more efficient methods to characterize viable parameter spaces. Second, each model has to be evaluated individually, which limits the number of models that can be evaluated. However, with the distribution of 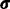 guiding model identification, topological augmentation can reduce the number of candidate models. Third, characterizing noise in complex biochemical systems with multiple variables can be difficult. The organization and geometry of viable parameter spaces may prevent identification of a single best model improvement. In this case, one can iteratively evaluate additional or modified reaction terms, based on biological knowledge and on information from the distribution of 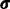 in a viable space. Fourth, topological augmentation may not be applicable to systems with much inherent noise (e.g. involving molecules with a low copy number). It is impossible to separate such noise from topological uncertainty. Finally, two steps of topological augmentation are currently not automatized and require some degree of human expertise and judgment in the execution. In step 5, we visually inspect the parameter space to identify non-negligible system noise terms (entries of 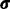). However, this step could be automatized with the topological filtering method proposed in Sunnåker *et al.* (2013b), which uses a parameter space exploration in combination with investigations of the effect of eliminated parameters. By identification of essential system noise parameters, it is possible to point to model parts with a potential for improvements in an automated fashion. In (the optional) step 6 of topological augmentation, additional terms are added to the SDEs, and a (potentially non-unique) mapping of the inferred temporal profiles of the unknown mechanisms to new ODE model terms is applied. It is important to keep in mind that the selected reaction terms must have a biological interpretation, and one approach would therefore be to construct a dictionary with biologically feasible reactions. Only terms that are specified in the dictionary are then candidates for incorporation into the model, e.g. based on symbolic regression (Schmidt *et al.*, 2011).

These limitations notwithstanding, topological augmentation can help to construct models systematically and rigorously in biochemistry and systems biology, but not only in these fields. It can be applied to infer the structure of any model based on ODEs.

## Supplementary Material

Supplementary Data
