# Supplementary material for: Topological augmentation to infer hidden processes in biological systems
Source: Bioinformatics. 2013 Dec 2;30(2):221–7. doi: 10.1093/bioinformatics/btt638 (PMC3892687; doi:10.1093/bioinformatics/btt638)
Supplement: Supplementary Data [file supp_30_2_221__index.html]

Topological Augmentation to Infer Hidden Processes in Biological Systems — Topological augmentation to infer hidden processes in biological systems — Topological augmentation to infer hidden processes in biological systems — Supplementary Data 

# Topological augmentation to infer hidden processes in biological systems

## Supplementary Data

files

**Files in this Data Supplement:**

- Supplementary Data - pdf file
