# Supplementary material for: Topological augmentation to infer hidden processes in biological systems
Source: Bioinformatics. 2013 Dec 2;30(2):221–7. doi: 10.1093/bioinformatics/btt638 (PMC3892687; doi:10.1093/bioinformatics/btt638)
Supplement: Supplementary Data [file supp_btt638_SuppData_Final.pdf]

Supplementary Data - Topological Augmentation to Infer Hidden Processes in Biological Systems

Mikael Sunnaker, Elias Zamora-Sillero, Adrián López García de Lomana,
Florian Rudroff, Uwe Sauer, Joerg Stelling, Andreas Wagner

October 28, 2013

List of supplementary materials:

1. Evaluation of the likelihood function
2. The extended Kalman filter
3. Parameter viability criteria
4. Parameter sampling
5. Pharmacokinetic model
6. Glutamine uptake experiments
7. Alternative models for glutamine transport
8. Gap1 regulation

1 Evaluation of the likelihood function

In Bayesian statistics, the Bayes theorem constitutes a mean to update the belief state about a hypothesis (e.g., the value of a parameter, or the plausibility of a model) due to new experimental observations. The belief state, before the new data is available, is conventionally referred to as the prior distribution, whereas the belief state after incorporation of the data is referred to as the posterior distribution. The posterior distribution is linked to the prior distribution, by the Bayes theorem, through the likelihood function, which represents the probability of the data given the hypothesis and a normalizing constant, called the evidence or marginal likelihood.

Assume that a set of observational data $\mathcal{Y} = [\mathbf{Y}_1, \ldots, \mathbf{Y}_K]$ is available, where the subscript denotes the time point index. The posterior distribution of a parameter point $\boldsymbol{\rho} = \{\boldsymbol{\theta}, \boldsymbol{\sigma}, \mathbf{S}_1, \ldots, \mathbf{S}_K]^T$, given $\mathcal{Y}$, is:

$$p(\boldsymbol{\rho}|\mathcal{Y}) = \frac{p(\mathcal{Y}|\boldsymbol{\rho})p(\boldsymbol{\rho})}{p(\mathcal{Y})}, \tag{1}$$

where $p(\mathcal{Y}|\boldsymbol{\rho})$ is the likelihood function, $p(\boldsymbol{\rho})$ is the prior distribution, and $p(\mathcal{Y})$ is the evidence (marginal likelihood). Following the approach of Overgaard *et al.*, 2005, we computed the likelihood of each parameter point from a quasi-likelihood function, for which the conditional densities are approximated by Gaussian densities. The likelihood function takes the form:

$$p(\mathcal{Y}|\boldsymbol{\rho}) = p(\mathbf{Y}_K, \mathbf{Y}_{K-1}, \ldots, \mathbf{Y}_1|\boldsymbol{\rho}) = \prod_{k=2}^{K} \big(p(\mathbf{y}_k|\mathbf{y}_{k-1}, \boldsymbol{\rho})\big)p(\mathbf{y}_1, \boldsymbol{\rho}) = \prod_{k=1}^{K} \frac{e^{-\frac{1}{2}\boldsymbol{\epsilon}_k^T \mathbf{R}_{(k|k-1)}^{-1} \boldsymbol{\epsilon}_k}}{\sqrt{|2\pi \mathbf{R}_{(k|k-1)}|}}, \tag{2}$$

with the residual at time point k: $\boldsymbol{\epsilon}_k = \mathbf{Y}_k - \mathbf{y}_{k|k-1}$. The predicted output and the corresponding covariance matrix at time point k, given all available data until time point $k-1$, are denoted by:

$$\mathbf{y}_{(k|k-1)} = \mathrm{E}[\mathbf{y}_k|\mathcal{Y}_{k-1}, \boldsymbol{\rho}], \tag{3}$$

and

$$\mathbf{R}_{(k|k-1)} = \mathrm{Var}[\mathbf{y}_k|\mathcal{Y}_{k-1}, \boldsymbol{\rho}], \tag{4}$$

respectively. To compute the likelihood we need to evaluate the terms $\mathbf{y}_{k|k-1}$ and $\mathbf{R}_{(k|k-1)}$, with a method capable of incorporating $\boldsymbol{\sigma}$ and $\mathbf{S}_1, \ldots, \mathbf{S}_K$. The extended Kalman filter has been used for this purpose (Overgaard *et al.*, 2005), where the term *extended* refers to the extended applicability of the Kalman filter to nonlinear models (Kalman, 1960). See Appendix 2 for details about the extended Kalman filter with SDEs, and how the different parameters are incorporated.

For practical purposes we define the following cost function:

$$E(\boldsymbol{\rho}|\mathcal{Y}) = -\ln[p(\mathcal{Y}|\boldsymbol{\rho})], \tag{5}$$

which returns the smallest cost for the most plausible parameter point.

2 The extended Kalman filter

Consider a SDE based model as in Eq. 3 of the main text, with the same output as in Eq. 1 of the main text:

$$M(\boldsymbol{\theta}) = \begin{cases} d\mathbf{x}(t) = f(\mathbf{x}, \mathbf{u}, \boldsymbol{\theta})dt + \boldsymbol{\sigma} d\boldsymbol{\omega}, \\ \mathbf{y}_k = \mathbf{h}\big(\mathbf{x}(t_k)\big) + \mathbf{e}_k, \mathbf{e}_k \sim \mathcal{N}(0, \mathbf{S}_k), \end{cases} \tag{6}$$

As disussed in Section 1 it is necessary to compute $\mathbf{y}_{(k|k-1)}$ and $\mathbf{R}_{(k|k-1)}$, where the subscript denotes timepoint k conditioned on data to time point $k-1$, to evaluate the cost function for a given parameter point. To do so we use an extended Kalman filter (EKF) following the approach of Kristensen *et al.*, 2005, which iteratively computes the required entities given a sequence of experimental data at K time points: $\mathcal{Y} = [\mathbf{Y}_1, \ldots, \mathbf{Y}_\mathrm{K}]$. The EKF starts from an estimate, before any experimental data is available, of the state $\hat{\mathbf{x}}_{1|0} = \mathrm{E}[\mathbf{x}(t = t_0)]$ and the corresponding covariance $\mathbf{P}_{1|0} = \mathrm{Var}[\mathbf{x}(t = t_0)]$. For time points $k = 1, \ldots, K$ an iterative procedure between an update step in which new experimental data is incorporated, and a prediction step to the next measurement time point, is applied. In the first step of this procedure the predicted response and the covariance of the response is evaluated at the predicted state variables:

$$\hat{\mathbf{y}}_{k|k-1} = \mathbf{h}(\hat{\mathbf{x}}_{k|k-1}), \tag{7}$$

$$\mathbf{R}_{k|k-1} = \mathbf{C}_k \mathbf{P}_{k|k-1} \mathbf{C}_k^T + \mathbf{S}_k, \tag{8}$$

where $\mathbf{C}_k$ is the Jacobian of $\mathbf{h}(\cdot)$ with respect to x_k evaluated at $\hat{\mathbf{x}}_{k|k-1}$. In the second step the Kalman gain $\mathbf{K}_k$ is computed:

$$\mathbf{K}_k = \mathbf{P}_{k|k-1} \mathbf{C}_k^T \mathbf{R}_{k|k-1}^{-1}, \tag{9}$$

which is used to update the estimate of $\mathbf{x}_k$ and $\mathbf{P}_k$ incorporating the data at time point k; $\mathbf{Y}_k$:

$$\hat{\mathbf{x}}_{k|k} = \hat{\mathbf{x}}_{k|k-1} + \mathbf{K}_k \boldsymbol{\epsilon}_k, \tag{10}$$

$$\mathbf{P}_{k|k} = \mathbf{P}_{k|k-1} - \mathbf{K}_k \mathbf{R}_{k|k-1} \mathbf{K}_k^T. \tag{11}$$

In the third step of the EKF the state and covariance is predicted at the next experimental readout time point:

$$\frac{d\hat{\mathbf{x}}_{t|k}}{dt} = f\left(\hat{\mathbf{x}}_{t|k}, \mathbf{u}(t), t, \boldsymbol{\theta}\right), \tag{12}$$

$$\frac{d\mathbf{P}_{t|k}}{dt} = \mathbf{A}_t \mathbf{P}_{t|k} + \mathbf{P}_{t|k} \mathbf{A}_t^T + \boldsymbol{\sigma}_t \boldsymbol{\sigma}_t^T, \tag{13}$$

where $\mathbf{A}_t$ is the Jacobian of $\mathbf{f}(\cdot)$ with respect to $\mathbf{x}_k$ evaluated at $\hat{\mathbf{x}}_{t|k}$ and $t \in [t_k, t_{k+1}]$. Eqs. 7- 13 constitute the EKF and are repeated for all time points to compute $\mathbf{x}_{k|k-1}$ and $\mathbf{P}_{k|k-1}$, $k = 1, \ldots, K$.

The initial condition for the pharmacokinetic model were set to $Q(0) = 5$ and $C(0) = 0$ and the uncertainty in the initally conditions $P_{1|0}$ was estimated by summing up the noise in the Wiener process between the first two experimental sampling time points:

$$P_{1|0} = \int_{t_0}^{t_1} e^{\mathbf{A}_s s} \boldsymbol{\sigma}_s \boldsymbol{\sigma}_s^T (e^{\mathbf{A}_s s})^T ds, \tag{14}$$

which follows the proposed approach of Kristensen *et al.*, 2005. In general this estimate may be multiplied by a scaling factor ≥ 1. For the glutamine models we set the initial condition, $\mathbf{x}_{1|0}$, to the experimentally measured values of the state variables. We also decided to estimate the uncertainty in the initial conditions directly by incorporating an additional parameter ψ so that $\mathbf{P}_{1|0} = \psi \mathbf{x}_{1|0}$, where $\psi \in [0, 0.2]$.

A complication for the application of the EKF to the glutamine models is that the compartmental volumes of the two state variables (external glutamine Q and a single cell C) are very different. In fact, the cell volume is 14 orders of magnitude smaller than the volume for the external medium. This means that for each iteration of the EKF in which new experimental data is incorporated and state variable Q is updated, the corresponding change in state variable C is 14 orders of magnitude as large. This phenomenon can be observed directly in the Jacobian matrix A_t. For this reason, we initially observed that all viable parameter points had very low values in $\boldsymbol{\sigma}$. This means that the magnitude of the entries of $\boldsymbol{\sigma}$ cannot be used as indicators for which parts of the model that may be improved. To get around this problem, we noted that the solution of the SDE for Q is independent of the SDE for C, since the reaction from Q to C is not reversible. For each parameter point that is evaluated we then first applied the EKF to the first equation only. In the second step we then applied the EKF to the full model, but with $\sigma_1 = 0$, so that the solution for Q is deterministic and the incorporation of each new data point leads to updates in C only and not in Q. Note that the rest of the parameters are the same both times we run the EKF. Finally, we used the residuals (and the corresponding variances) for Q from the first time we run the filter together with the residuals (and the corresponding variances) for C from the second run of the EKF. This efficiently allows us to use σ_1 as an indicator of how the first equation may be improved, which is separated from σ_2, as an indicator for the second equation. Finally, note that this is not an issue for the pharmacokinetic model for which the compartmental volumes are of the same order of magnitude.

3 Parameter viability criterium

From the cost function $E(\boldsymbol{\rho}|\mathcal{Y})$, we computed a *viability threshold* $E(\boldsymbol{\rho}|\mathcal{Y})_v$ under the assumption of Gaussian distributed residuals (difference between experimental data and model predictions) (Sunnåker *et al.*, 2013). The viability threshold is based on the existence of a model M^* and an unknown parameter point $\boldsymbol{\theta}^*$ with the same data generating characteristics as the underlying biochemical system. Given a set of experimental data generated by the system, the viability threshold equals the expected likelihood plus α standard deviations ($\alpha = 2$ in this article):

$$E(\boldsymbol{\theta}, \mathcal{Y}, M_i) \leq \ln\left(e^{\alpha\sqrt{\frac{\beta}{2}}} \sqrt{(2\pi e)^{\beta} |\mathbf{S}|}\right) \tag{15}$$

where parameter point $\boldsymbol{\theta}$ is viable in model M_i if Eq. (15) is satisfied, β is the number of data points, and S is the diagonal covariance matrix for the measurement noise (for details see Sunnåker *et al.* (2013)). If there is risk for overfitting (the number of parameters is large in comparison to the amount of data points) it may in addition be relevant introduce a lower log-likelihood threshold equal to the expected log-likelihood minus two standard deviations:

$$E(\boldsymbol{\theta}, \mathcal{Y}, M_i) \geq \ln\left(e^{-\alpha\sqrt{\frac{\beta}{2}}} \sqrt{(2\pi e)^{\beta} |\mathbf{S}|}\right) \tag{16}$$

However, in this article we use the viability cutoff to identify the parameter space regions of high likelihood, so the lower threshold is not necessary. We were also not able to identify parameter points below the lower threshold for any of the models analyzed in this article. The viability threshold can alternatively also be computed with bootstrapping of a multivariate normal distribution with zero mean and the same standard deviation as $\mathcal{Y}$.

However, this threshold is not valid in SDE based models, since we cannot expect the difference between model predictions and the experimenal data to be Gaussian distributed due to the inherent model stochasticity (system noise). The system noise

causes the model to produce different predictions each time the model is simulated, and the predicted trajectories of state variables are correlated over time. Since the measurement data is given, the distribution of the residuals (at the observation time points) will therefore not be Gaussian distributed, but biased by the particular simulation results. For the SDE models we therefore used other heuristic approaches to compute a viability threshold. In the case of the PK/PD models we used a Metropolis Monte-Carlo algorithm, which was run until the generated parameter values reached a stationary distribution. We then set the viability threshold equal to the mean of the stationary distribution plus two standard deviations. For the glutamine transport models this turned out to be impractical since the standard deviations of the stationary distribution was very small, which resulted in that only a region very close to the most likely parameter point was explored. Instead, we defined a viability threshold for which the difference in likelihood to the most likely parameter point is large. The viability threshold is chosen so that the contribution of inviable parameter points in the inference process is close to negligible.

It is also informative to study the form of the viable space and in particular the projection of the viable parameter space to the $\boldsymbol{\sigma}$ parameters, as the viability threshold is successively lowered. This procedure helped us to quantify the improvements due to incorporation of model extensions, since the simpler models and corresponding likelihood values can be obtained through elimination of some of the model parameters, for the models investigated in this paper. Therefore the viable space of the simpler model will be a subspace of the viable space of the improved model when the viability threshold is sufficiently low.

4 Parameter sampling method

For the exploration of the parameter space we employ the method described by Zamora-Sillero *et al.*, 2011, which is based on an out-of-equlibrium Metropolis Monte-Carlo algorithm, in combination with a refined search of the identified regions using ellipsoid expansions. Starting from a parameter point within the parameter bounds, new points are successively drawn from a Gaussian distribution, and accepted either if the negative log-likelihood value of the new point x_{new} is smaller than the previous point x_{old}, or otherwise compared to the Boltzmann distribution

$$\exp(-\beta(x_{\text{old}} - x_{\text{new}})) > r, \tag{17}$$

where r is a random variable drawn from a uniform distribution on the unit interval, and $\beta \in \mathbb{R}_+$ corresponds to the thermodynamic beta ($\beta = 1/(k_B T)$, where k_B is the Boltzmann constant and T is the temperature). To increase the rate of convergence of the sampling algorithm the covariance matrix of the Gaussian distribution is increased if the acceptance rate of new parameter points is high, and decreased if the acceptance rate is low. The identified viable regions are then explored in greater detail by successively expanding ellipsoids to cover the viable parameter region. As a rule of thumb the number of ellipsoids is equal to the dimension of the parameter space. Finally, the viable regions characterized in the first two steps are uniformly sampled, and these parameter points are used to make model predictions and to compare models.

5 Pharmacokinetic model - Setting

Simulation time is set to $t \in [0, 300]$ (min) and the parameter values are set to $Q(t = 0) = 5$ mg, $C(t = 0) = 0$ mg/L, $C_L = 0.05$ L/min, $V = 5$ l, $V_{\max} = 0.1$ mg/min, and $K_{\text{M}} = 0.5$ mg as in (Kristensen *et al.*, 2005). The proportional measurement error model takes the form $y_k = C_k(1 + 0.01e_k), e_k \sim \mathcal{N}(0, 1), k = 1, \ldots, K$. A modest measurement error (e.g., $\tilde{e} = 0.01e_k$) means that the following approximation holds: $\tilde{y}_k = log(y_k) = log(C_k(1 + \tilde{e}_k)) = log(C_k) + log(1 + \tilde{e}_k) \approx log(C_k) + \tilde{e}_k$, where $\tilde{e}_k \sim \mathcal{N}(0, S_k)$ and a Maclaurin expansion to the first term was used in the last step. The $N = 20$ artificial experimental data are generated at time points: 1, 2, 4, 6, 8, 10, 15, 20, 30, 40, 50, 60, 70, 80, 90, 100, 150, 200, 250, and 300 (min).

The pharmacokinetic ODE models take the form:

$$\begin{pmatrix} dQ \\ dC \end{pmatrix} = \begin{pmatrix} 1 & 0 \\ 0 & \frac{1}{V} \end{pmatrix} \begin{pmatrix} -1 & 0 \\ 1 & -1 \end{pmatrix} \begin{pmatrix} r_1 \\ r_2 \end{pmatrix} dt + \begin{pmatrix} \sigma_1 & 0 \\ -\frac{1}{V}\sigma_1 & \sigma_2 \end{pmatrix} \begin{pmatrix} d\omega_t^1 \\ d\omega_t^2 \end{pmatrix}. \tag{18}$$

where the reactions in the linear model take the form: $r_1 = k_{\text{A}} Q$ and $r_2 = C_L C$. For the nonlinear SDE model reaction r_2 is the same and: $r_1 = \frac{V_{max} Q}{K_M + Q}$.

We explored each of the parameters in the different models in the region $[-4, 4]$ (log-scale), with the viability condition established by an initial run of the Metropolis Monte Carlo algorithm. The theoretical viability threshold for ODE models, given the artificial data, is: $E(\boldsymbol{\rho}|\mathcal{Y})_v = -57.40$ (we obtained $E(\boldsymbol{\rho}|\mathcal{Y})_v = -53.51$ with the MCMC approach for the MM model). For the linear SDE model the viability threshold of the cost function: $E(\boldsymbol{\rho}|\mathcal{Y})_v = -12.86$, which corresponds to the mean of the stationary distribution obtained in the Monte Carlo exploration plus two standard deviations (mean: $\langle E(\boldsymbol{\rho}|\mathcal{Y}) \rangle = -16.84$, std: $\sigma_{E(\boldsymbol{\rho}|\mathcal{Y})} = 1.99$). The viable parameter points for the linear SDE model are shown in Fig. S2. The viability threshold for the MM model was established with the same procedure; $E(\boldsymbol{\rho}|\mathcal{Y})_v = -53.51$ ($\langle E(\boldsymbol{\rho}|\mathcal{Y}) \rangle = -60.14$ and $\sigma_{E(\boldsymbol{\rho}|\mathcal{Y})} = 3.31$). For the MM-model with $C_L = 1$ the viability threshold is: 11.56 ($\langle E(\boldsymbol{\rho}|\mathcal{Y}) \rangle = 9.25$ and $\sigma_{E(\boldsymbol{\rho}|\mathcal{Y})} = 1.15$). Finally, we also analyzed the model for another data set with $y_k = C_k(1 + 0.1e_k)$, which gives very similar results Fig. S3.

6 Experimental setting

6.1 Cultivation

Experiments were performed with the prototrophic *Saccharomyces cerevisiae* strain YSBN6 [MATa FY3 ho::HphMX4] (Canelas *et al.*, 2010). Cells were taken from a frozen glycerol stock and plated on a YPD agar plate. A preculture was prepared by inoculating a single colony of *S. cerevisiae* YSBN6 into a 500 mL shakeflask containing 50 mL of liquid yeast minimal mineral medium supplemented with 20 g/L of glucose and incubated at 30°C in a rotary shaker at 300 rpm for 24 hours. From precultures we inoculated 25 mL of medium in a 500 mL shakeflask to a starting optical density at 600 nm (OD_{600}) of 0.03 – 0.08 for the glutamine depletion experiment.

All cultivations were done in Yeast minimal medium based on the approach of Blank and Sauer, 2004 with minor modifications. The base medium contained the following components (per Liter): 5g K_2SO_4, 3g KH_2PO_4, 0.5g $MgSO_4$•$7H_2O$, 15mg EDTA, 4.5mg $ZnSO_4$•$7H_2O$, 0.3mg $CoCl_2$•$6H_2O$, 1.0mg $MnCl_2$•$4H_2O$, 0.3mg $CuSO_4$•$5H_2O$, 4.5mg $CaCl_2$•$2H_2O$, 3.0mg $FeSO_4$•$7H_2O$, 0.4mg $NaMoO_4$•$2H_2O$, 1.0mg H_3BO_3, 0.1mg KI, 0.05mg biotin, 1.0mg calcium pantothenate, 1.0mg nicotinic acid, 25mg inositol, 1.0mg pyridoxine, 0.2mg p-aminobenzoic acid, 1.0mg thiamin, and 10 mM potassium hydrogen phthalate buffer (pH 5). The base medium was supplemented with 20 g/L D-glucose and 1 g/L of L-glutamine for precultures and with 20 g/L D-glucose, 500 mg/L of L-glutamine, and 250 mg/L L-proline for the glutamine to proline downshift experiment.

6.2 Glutamine to proline down-shift experiment (Gln→Pro)

Throughout independent triplicate experiments we monitored cell growth (OD600), extracellular concentrations of glutamine, proline, glucose and ethanol, and intracellular concentrations of glutamine (the data of a representative experiment is shown in Fig. S12). To assess the time of the actual shift from glutamine to proline consumption that we wanted to follow with higher sampling density, we semi-quantitatively determined relative concentrations of glutamine in the broth by at-line direct flow injection mass spectrometry analysis on an Agilent LC-qTOF system, allowing readouts within less than 2 minutes after sampling (Fuhrer *et al.*, 2011). The time at which most glutamine had been consumed and arrived to a plateau of significantly lower concentration is referred to as the time of shift (t_0), and we increased the sampling frequency around this time point. For the sake of consistency the data time series from the three independent experiments were aligned in time, with t_0 as the reference point. Standard deviations were computed for the temporally aligned glutamine measurements, which are shown in Fig. 3A (blue dots). Initial concentrations of intracellular glutamine were mapped to literature reported values (Gancedo and Gancedo, 1973; van Riel,N.A. *et al.*, 2000). At the time of shift, the culture had reached a density of 0.81 ± 0.06 OD_{600} units and the maximum specific growth rate declined from 0.36 ± 0.01 h^{-1} before the shift to 0.24 ±0.03 h^{-1} after the shift, reflecting the shift nitrogen source quality from glutamine to proline.

6.3 Metabolite quantification

Intracellular glutamine concentrations were determined by LC-MS/MS as described by Buescher *et al.*, 2010 and extracellular concentrations of glucose and ethanol were determined by HPLC as described elsewhere (Heer and Sauer, 2008). Extracellular concentrations of glutamine and proline were determined by GC-MS after derivatization. For this purpose, cells were separated by centrifugation and the supernatant was derivatized according to an adapted protocol from Zamboni *et al.*, 2009. Specifically, 10 μL of broth supernatant was mixed with 10 μL internal standard (0.5 μM norvaline, 0.5 μM glutarate) (Buescher *et al.*, 2009), this mixture was dried to complete dryness in a vacuum centrifuge and then resuspended in 20 μL dry DMF (Sigma-Aldrich). Derivatization was accomplished by incubation at 85°C for 1h upon addition of 20 μL of TBDMS (N-(tert-butyldimethylsilyl)-N-methyl-trifluoroacetamide with 1% tert-butyldimethyl-chlorosilane, Sigma-Aldrich). Specific fragments for glutamine and proline (m/z: 431 and 258, respectively) were recorded using a 6890N GC system (Agilent Technologies) combined with a 5875 Inert XL MS system (Agilent Technologies) in electron impact mode and a scan range between 150 amu and 550 amu. Quantification was achieved by using external standards consisting of the pure substance obtained from Sigma.

7 Alternative models for glutamine transport

The symbols and parameter bounds in the glutamine uptake models are presented in Table 1.

7.1 Michaelis-Menten transport model

The model based on Michaelis-Menten (MM) kinetics, and referred to as the MM model, is defined as:

$$\begin{aligned} \dot{Q} &= -U\,\frac{1}{V_{\mathrm{f}}}\,\frac{V_{\max}^{\mathrm{T}_1}\,Q}{K_{\mathrm{M}}^{\mathrm{T}_1}+Q}, \\ \dot{C} &= \frac{1}{V_{\mathrm{c}}}\,\frac{V_{\max}^{\mathrm{T}_1}\,Q}{K_{\mathrm{M}}^{\mathrm{T}_1}+Q} - D\,C, \end{aligned} \tag{19}$$

where Q is the glutamine concentration in the medium and C is the glutamine concentration in the cells, and $Q(0) = 0.0073$ and $C(0) = 7.2910^{-3}$ based on estimates from the experimental data. V_f and V_c refer to the flask and the cell volume, respectively. U is an input function for the number of cells (see Fig. S4). The structural parameter D is the glutamine consumption by the cell, $V_{\rm max}^{\rm T_1}$ is the maximum rate of glutamine transport and $K_{\rm M}^{\rm T_1}$ is the concentration of external glutamine for which the transport rate is half-maximal. We found viable parameter points by running several instances of the local optimization algorithm. The viable parameter space was then characterized (Fig. S5).

Although we could find viable parameter points for the MM model, the residuals of the extracellular glutamine concentration are correlated over time (i.e., the model predicts a too low concentration of external glutamine at most time points, see Fig. 3A of the main text. To investigate if, and how, the model could be improved we reformulated the ODEs-based model into a model based on SDEs:

$$\begin{aligned} dQ &= \left(-U\ \frac{1}{V_{\rm f}}\ \frac{V_{\rm max}^{\rm T_1}\ Q}{K_{\rm M}^{\rm T_1} + Q} \right) dt + \sigma_1 d\omega_1, \\ dC &= \left(\frac{1}{V_{\rm c}}\ \frac{V_{\rm max}^{\rm T_1}\ Q}{K_{\rm M}^{\rm T_1} + Q} - D\ C \right) dt + \sigma_2 d\omega_2, \end{aligned} \tag{20}$$

where ω_1 and ω_2 are Wiener processes. We next explored the parameter space of the MM model (the investigated parameters are defined in Table 2). The projection of the viable parameters into $\boldsymbol{\sigma}$ is shown in Fig. 3B of the main text. While the distribution of viable parameter values for σ_2 shows that the second equation sufficiently well describes the correct reaction mechanism, the distribution of viable values for σ_1 shows that equation one may be improved.

In order to investigate how the MM model may be improved we created an extended version of the model. In this model we have included an additional reaction mechanism for the transport of glutamine into the cells, with a reaction rate that is proportional to Q. The full extended model takes the form:

$$\begin{aligned} dQ &= \frac{-U}{V_{\rm f}} \left(\frac{V_{\rm max}^{\rm T_1}\ Q}{K_{\rm M}^{\rm T_1} + Q} + KWQ \right) dt + \sigma_1 d\omega_1, \\ dC &= \left(\frac{1}{V_{\rm c}} \left(\frac{V_{\rm max}^{\rm T_1}\ Q}{K_{\rm M}^{\rm T_1} + Q} + KWQ \right) - D\ C \right) dt + \sigma_2 d\omega_2, \\ dW &= \sigma_3 d\omega_3, \end{aligned} \tag{21}$$

where ω_3 is a Wiener process, and $W(0) = 0$. The variable K has the property that $K = 0$ at $t = 0$ and that $K = 1$ when $Q < \tau_a$. Note that the experimental data indicates that Q decreases over time. The unknown parameter τ_a is estimated together with the structural parameters and $\boldsymbol{\sigma}$. The EKF is used to predict the value of state variable W over time, which indicates the form of the additional hypothetical reaction mechanism. The parameter space of this model was explored and the result is shown in Fig. 4A of the main text. Interestingly the method predicts that W stays small until $t \sim 15 - 20$ and then increases rapidly as glutamine is depleted in the external medium. We also observed a gradual increase in W the for the last 100 minutes. To incorporate these observations in the model inference process we propose the regulated Michaelis-Menten model, which is described in the next section.

7.2 Regulated Michaelis-Menten Model

Two of the four glutamine permeases in yeast, Gap1 and Gnp1, are reported to be under NCR control (Risinger *et al.*, 2006; Schreve *et al.*, 1998). From the results of the experiments by Risinger *et al.*, 2006, we constructed an exponential model, $g(t)$, for the NCR adaptation (Fig. S7). The regulated Michaelis-Menten (rMM) model takes the form:

$$\begin{aligned} \dot{Q} &= -U\ \frac{1}{V_{\rm f}} \left(\frac{V_{\rm max}^{\rm T_1}\ Q}{K_{\rm M}^{\rm T_1} + Q} + g(t)\ K\ \frac{V_{\rm max}^{\rm T_2}\ Q}{K_{\rm M}^{\rm T_2} + Q} \right), \\ \dot{C} &= \frac{1}{V_{\rm c}} \left(\frac{V_{\rm max}^{\rm T_1}\ Q}{K_{\rm M}^{\rm T_1} + Q} + g(t)\ K\ \frac{V_{\rm max}^{\rm T_2}\ Q}{K_{\rm M}^{\rm T_2} + Q} \right) - D\ C, \end{aligned} \tag{22}$$

where the parameters also used in the MM model have the same interpretation as here; $V_{\rm max}^{\rm T_2}$ refers to the maximum rate of glutamine transport by the second permease, and $K_{\rm M}^{\rm T_2}$ is the concentration of external glutamine for which the transport rate is half-maximal.

7.3 Extensions of the rMM model

Given the literature evidence of the presence of four different glutamine permeases (Gap1 (Risinger *et al.*, 2006), Gnp1 (Zhu *et al.*, 1996), Agp1 (Schreve *et al.*, 1998) and Dip5 (Regenberg *et al.*, 1998)), we extended the rMM model to three or four

MM terms. We defined model e1rMM as:

$$\begin{aligned}\dot{Q} &= -U\,\frac{1}{V_\mathrm{f}}\left(\frac{V_\mathrm{max}^{\mathrm{T}_1}\,Q}{K_\mathrm{M}^{\mathrm{T}_1}+Q} + g(t)\,K\,\frac{V_\mathrm{max}^{\mathrm{T}_2}\,Q}{K_\mathrm{M}^{\mathrm{T}_2}+Q} + \frac{V_\mathrm{max}^{\mathrm{T}_3}\,Q}{K_\mathrm{M}^{\mathrm{T}_3}+Q}\right),\\ \dot{C} &= \frac{1}{V_\mathrm{c}}\left(\frac{V_\mathrm{max}^{\mathrm{T}_1}\,Q}{K_\mathrm{M}^{\mathrm{T}_1}+Q} + g(t)\,K\,\frac{V_\mathrm{max}^{\mathrm{T}_2}\,Q}{K_\mathrm{M}^{\mathrm{T}_2}+Q} + \frac{V_\mathrm{max}^{\mathrm{T}_3}\,Q}{K_\mathrm{M}^{\mathrm{T}_3}+Q}\right) - D\,C,\end{aligned} \tag{23}$$

and e2rMM as:

$$\begin{aligned}\dot{Q} &= -U\,\frac{1}{V_\mathrm{f}}\left(\frac{V_\mathrm{max}^{\mathrm{T}_1}\,Q}{K_\mathrm{M}^{\mathrm{T}_1}+Q} + g(t)\,K\,\frac{V_\mathrm{max}^{\mathrm{T}_2}\,Q}{K_\mathrm{M}^{\mathrm{T}_2}+Q} + \frac{V_\mathrm{max}^{\mathrm{T}_3}\,Q}{K_\mathrm{M}^{\mathrm{T}_3}+Q} + g(t)\,K\,\frac{V_\mathrm{max}^{\mathrm{T}_4}\,Q}{K_\mathrm{M}^{\mathrm{T}_4}+Q}\right),\\ \dot{C} &= \frac{1}{V_\mathrm{c}}\left(\frac{V_\mathrm{max}^{\mathrm{T}_1}\,Q}{K_\mathrm{M}^{\mathrm{T}_1}+Q} + g(t)\,K\,\frac{V_\mathrm{max}^{\mathrm{T}_2}\,Q}{K_\mathrm{M}^{\mathrm{T}_2}+Q} + \frac{V_\mathrm{max}^{\mathrm{T}_3}\,Q}{K_\mathrm{M}^{\mathrm{T}_3}+Q} + g(t)\,K\,\frac{V_\mathrm{max}^{\mathrm{T}_4}\,Q}{K_\mathrm{M}^{\mathrm{T}_4}+Q}\right) - D\,C.\end{aligned} \tag{24}$$

where the parameters shared with the rMM model have the same interpretation in e1rMM and e2rMM. The parameters $V_\mathrm{max}^{\mathrm{T}_3}$ and $V_\mathrm{max}^{\mathrm{T}_4}$ represent the maximum rate of glutamine transport reactions by the third and fourth permeases, respectively. $K_\mathrm{M}^{\mathrm{T}_3}$ and $K_\mathrm{M}^{\mathrm{T}_4}$ are the the concentrations of external glutamine for which the corresponding transport rates are half-maximal.

8 Gap1 regulation

Risinger *et al.*, 2006, conducted quantitative experiments to investigate the reversible inactivation of the permease Gap1. Measurements were produced for an experiment in which the activity of Gap1 was monitored after amino acid depletion from the medium. The data from this experiment was used to fit a model for $g(t)$ (Fig. S7):

$$g(t) = 1 - C_0 e^{-kt}, \tag{25}$$

where t denotes time (min), C_0 was set to 1, and k was estimated to be $k = 0.0188$.

References

Berg,J.M. *et al.* (2002) *Biochemistry.* 5th edn. W.H. Freemann.

Blank,L.M. and Sauer,U. (2003) TCA cycle activity in Saccharomyces cerevisiae is a function of the environmentally determined specific growth and glucose uptake rates. *Microbiology*, **150**, 1085–1093.

Buescher, J.M. (2009) Cross-platform comparison of methods for quantitative metabolomics of primary metabolism. *Anal. Chem.*, **81**, 2135–2143.

Buescher, J.M. *et al.* (2010) Ultrahigh performance liquid chromatography-tandem mass spectrometry method for fast and robust quantification of anionic and aromatic metabolites. *Anal. Chem.*, **82**, 4403–4412.

Canelas,A.B. *et al.* (2010) Integrated multilaboratory systems biology reveals differences in protein metabolism between two reference yeast strains. *Nat. Commun.*, **1**, 145.

Fuhrer,T. *et al.* (2011) High-throughput, accurate mass metabolome profiling of cellular extracts by flow injection-time-of-flight mass spectrometry. *Anal. Chem.*, **83**, 7074–7080.

Gancedo,J.M. and Gancedo,C. (1973) Concentrations of intermediary metabolites in yeast. *Biochimie*, **55**, 205–211.

Gatz,D. and Smith,L. (1995) The standard error of a weighted mean concentration–I. bootstrapping vs other methods. *Atmospheric Environment*, **29**, 1185–1193.

Heer,D. and Sauer,U. (2008) Identification of furfural as a key toxin in lignocellulosic hydrolysates and evolution of a tolerant yeast strain. *Microb. Biotechnol.*, **1**, 497–506.

Kalman,R.E. (1960) A New Approach to Linear Filtering and Prediction Problems. *Transactions of the ASME–Journal of Basic Engineering*, **82**, 35–45.

Kristensen,N.R. *et al.* (2005) Using stochastic differential equations for PK/PD model development. *J. Pharmacokinet. Pharmacodyn.*, **32**, 109–141.

Overgaard,R.V. *et al.* (2005) Non-linear mixed-effects models with stochastic differential equations: implementation of an estimation algorithm. *J. Pharmacokinet. Pharmacodyn.*, **32**, 85–107.

Regenberg,B. *et al.* (1998) Dip5p mediates high-affinity and high-capacity transport of L-glutamate and L-aspartate in Saccharomyces cerevisiae. *Curr. Genet.*, **33**, 171–177.

Risinger,A.L. *et al.* (2006) Activity-dependent reversible inactivation of the general amino acid permease. *Mol. Biol. Cell*, **17**, 4411–4419.

Schreve,J.L. *et al.* (1998) The Saccharomyces cerevisiae YCC5 (YCL025c) gene encodes an amino acid permease, Agp1, which transports asparagine and glutamine. *J. Bacteriol.*, **180**, 2556–2559.

Sunnåker,M. *et al.* (2013) Automatic Generation of Predictive Dynamic Models Reveals Nuclear Phosphorylation as the Key Msn2 Control Mechanism. *Sci. Signal.*, **6**:ra41.

van Riel,N.A. *et al.* (2000) Dynamic optimal control of homeostasis: an integrative system approach for modeling of the central nitrogen metabolism in Saccharomyces cerevisiae. *Metab. Eng.*, **2**, 49–68.

Zamboni,N. *et al.* (2009) (13)C-based metabolic flux analysis. *Nat. Protoc.*, **4**, 878–892.

Zamora-Sillero,E. *et al.* (2011) Efficient characterization of high-dimensional parameter spaces for systems biology. *BMC Syst. Biol.*, **5**:142.

Zhu,X. *et al.* (1996) GNP1, the high-affinity glutamine permease of S. cerevisiae. *Curr. Genet.*, **30**, 107–114.

SI Tables

Table 1: List of symbols used for the models.

Symbol	Description	Units
t	Time.	min
t_0 or $t = 0$	Time at which the metabolic shift starts.	min
	State variables	
Q	Concentration of glutamine in the media.	M
C	Concentration of glutamine in an average cell.	M
U	Number of cells (Supplementary Data, Figure 4).	cell
	Model parameters	
τ_a	Concentration threshold for activation of the regulated term.	$\mathrm{mol} \cdot \mathrm{L}^{-1}$
D	Consumption rate of Q in the cell.	min^{-1}
$V_{\mathrm{max}}^{\mathrm{T_i}}$	Maximum transport rate ($i = 1, \ldots, 4$).	$\mathrm{mol} \cdot \mathrm{L}^{-1} \cdot \mathrm{min}^{-1}$
$K_{\mathrm{M}}^{\mathrm{T_i}}$	Concentration of external glutamine at which transport rate is half-maximal ($i = 1, \ldots, 4$).	$\mathrm{mol} \cdot \mathrm{L}^{-1}$
	Constants	
V_{f}	Volume of the flask.	L
V_{c}	Volume of the cell.	L

Table 2: Model parameter ranges explored.

Parameter	Range	Motivation
τ_a	$(1\times10^{-5}$,$1\times10^{-2})$	It has been observed that 1 mM of glycine stops Gap1 activity (Risinger *et al.*, 2006). We explored a range of three orders of magnitude accounting for variability on amino acid response and because lowest measure values for Q, lay on the 1×10^{-4} M range .
D	$(1\times10^{-6}$,$1\times10^{0})$	A preliminary computational exploratory search between 1×10^{-6} and 1×10^{14} found viable values constrained around the 1×10^{-2} range. Higher values (around 1×10^{5}) resulted on problematic trajectories (data not shown), therefore exploration ranges were reduced to slower dynamical regimes, consistent with the biological context.
$K_{\mathrm{M}}^{\mathrm{T_i}}$	$(1\times10^{-7}$,$1\times10^{-1})$	Typical K_{M} values for enzymes have been described to range from 1×10^{-7} to 1×10^{-1} M (Berg *et al.*, 2002). We have explored the complete range, except for in specifically mentioned explorations reduced to the millimolar or micromolar range. In those cases, the ranges of K_M values used are $[1\times10^{-4}$,$1\times10^{-2}]$ and $[1\times10^{-7}$,$1\times10^{-5}]$, respectively.
$V_{\mathrm{max}}^{\mathrm{T_i}}$	$(1\times10^{-15}$,$1\times10^{5})$	V_{max} is defined as $V_{\mathrm{max}} \equiv k_{\mathrm{cat}}E$ (E is an enzyme). Typical k_{cat} values can be obtained from measured values of $k_{\mathrm{cat}}/K_{\mathrm{M}}$, which range from 1×10^{-1} to 1×10^{9} $\mathrm{M}^{-1}\mathrm{s}^{-1}$, therefore, k_{cat} takes the range from 1×10^{-8} to 1 $\times10^{8}$ min^{-1} (see above for range of K_M values). Typical E concentrations are in the μM range (giving V_{max} values in the range from 1×10^{-14} to 1×10^{2}), and we investigated a broad range of V_{max} values from 1×10^{-15} to 1×10^{5}.

Table 3: Posterior model probabilities for the linear (M_1-M_4), and the nonlinear (M_5-M_8), pharmacokinetic models. The posteriors were computed by uniformly sampling the high-likelihood parameter regions for each model, and with the viability threshold from the MCMC approach described in section 3.

Model	Eliminated parameters	Posterior probability
M_1	-	0
M_2	σ_1	0
M_3	σ_2	0
M_4	σ_1, σ_2	0
M_5	-	0.0083
M_6	σ_1	0.1467
M_7	σ_2	0.0478
M_8	σ_1, σ_2	0.7971

SI Figures

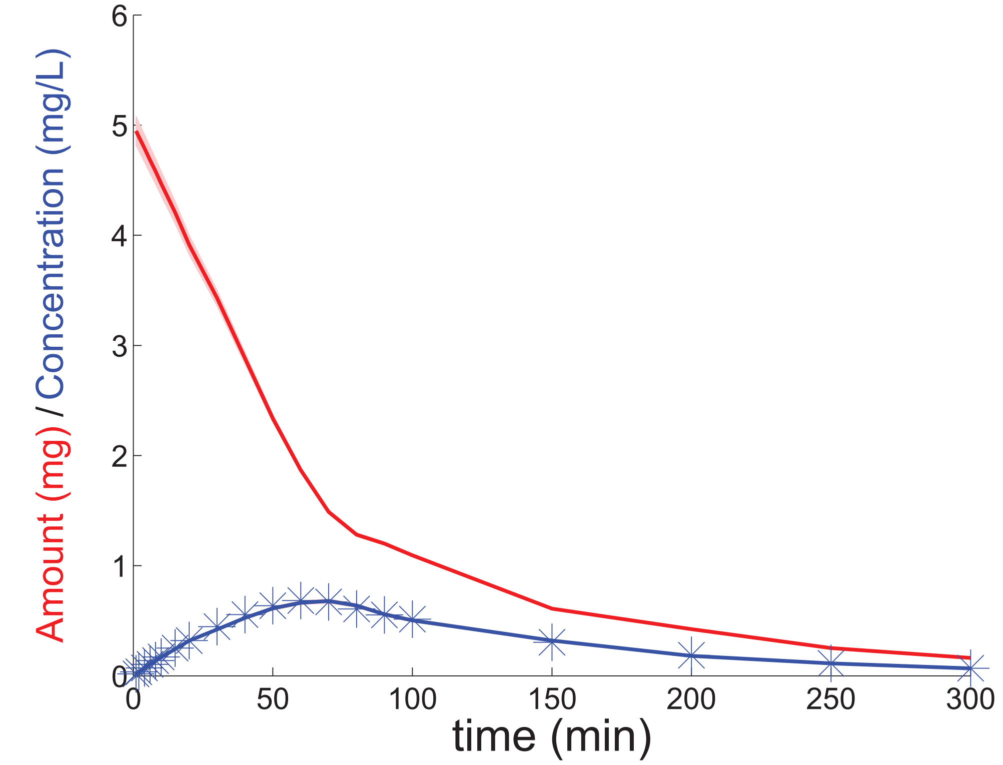

Figure 1: **Trajectories for the state variables Q (red) and C (blue) for the three states linear pharmacokinetic model.** Blue stars represent the *in silico* generated data for C.

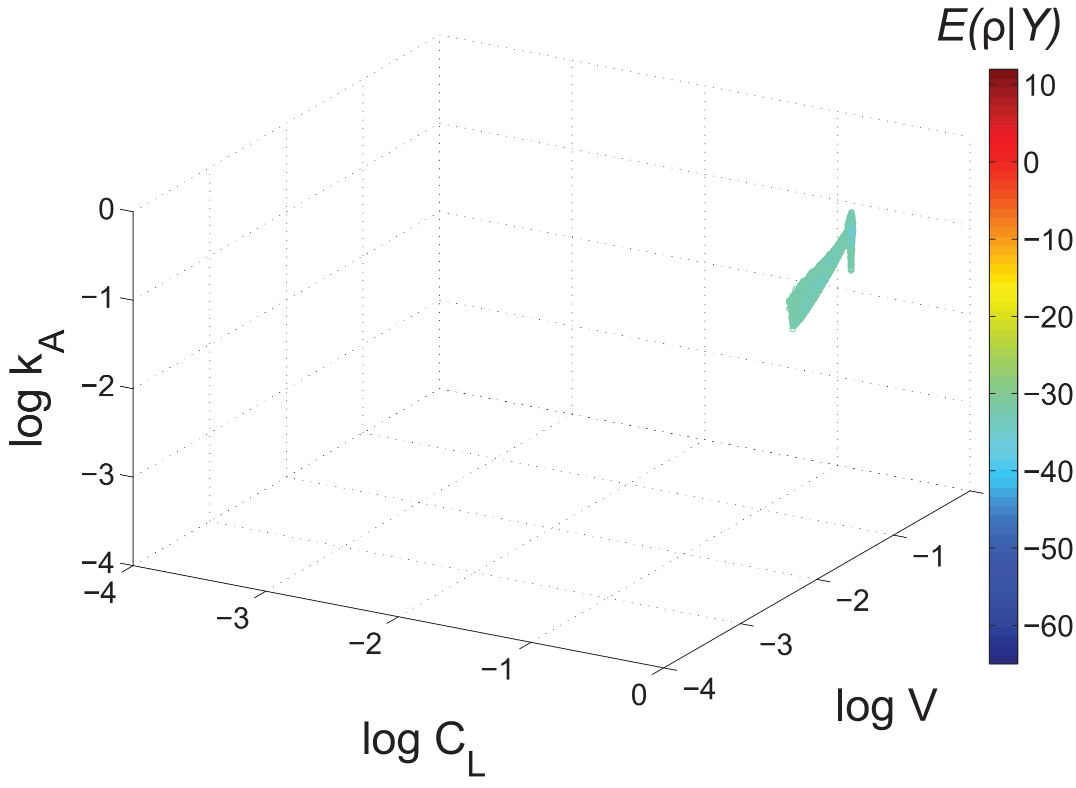

Figure 2: **Viable parameters of the linear pharmacokinetic model** Projection of the uniform distribution of viable parameter points into V, C_L, and k_A for the three states linear pharmacokinetic model.

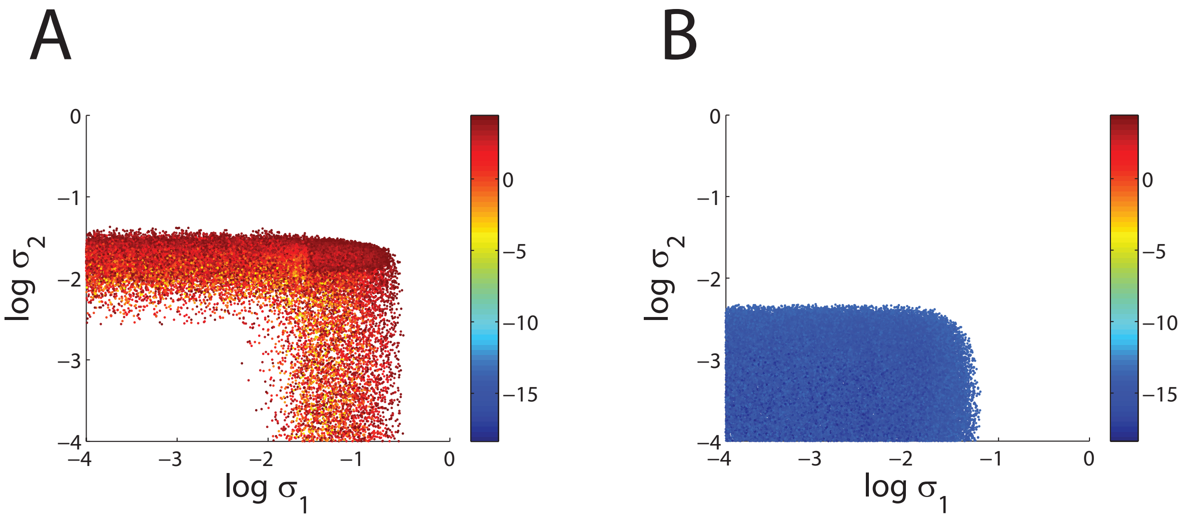

Figure 3: **Viable parameters of the linear and nonlinear pharmacokinetic models.** Projection of the uniform distribution of viable parameter points, with 10% multiplicative measurement noise, projected to σ_1 and σ_2 for **A**: the linear pharmacokinetic model, **B**: nonlinear pharmacokinetic model.

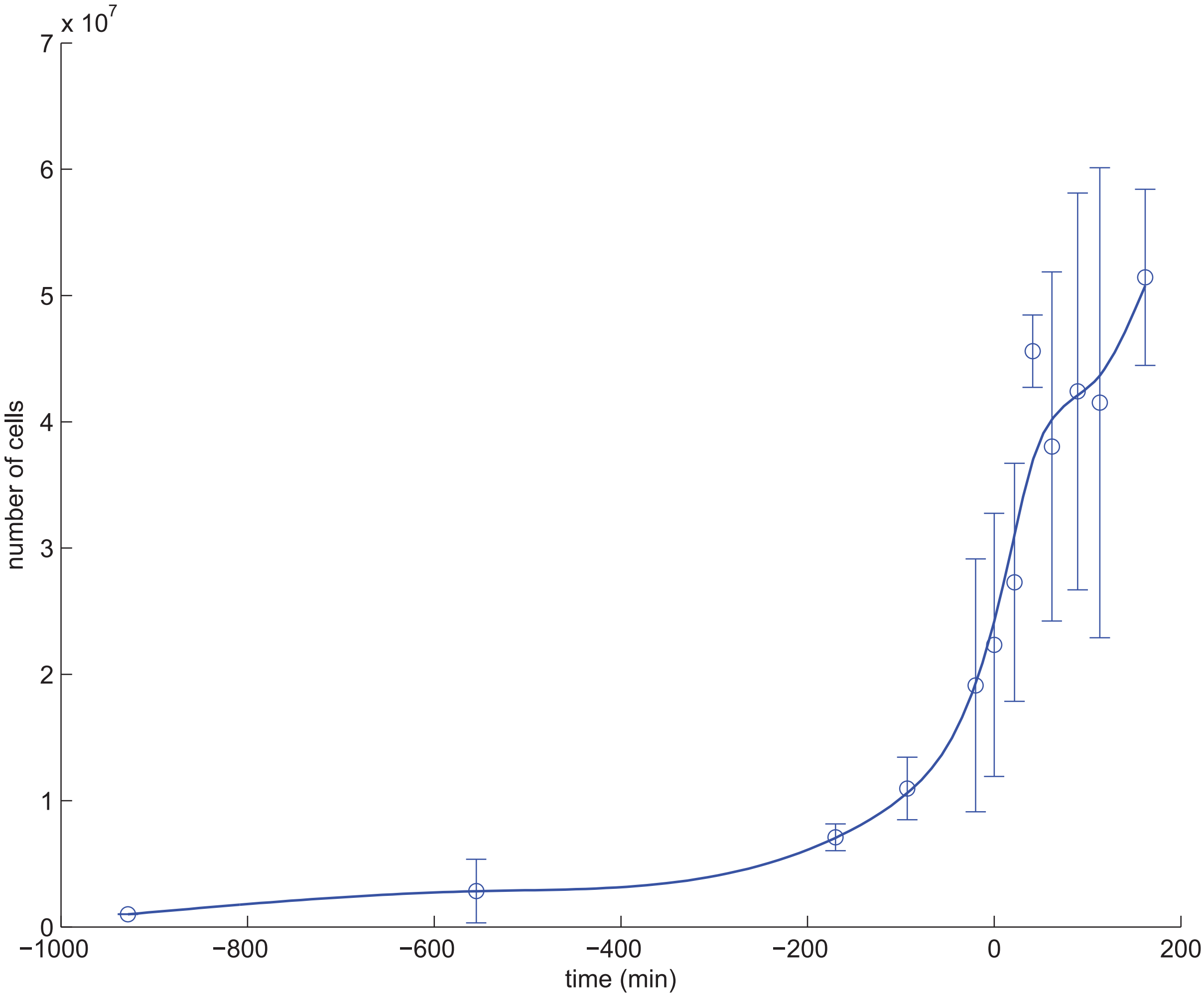

Figure 4: **Evolution of cell growth during the experiment.** Mean and standard deviation ($n = 3$) is shown. Solid blue line represents a cubic smoothing spline applied to the data.

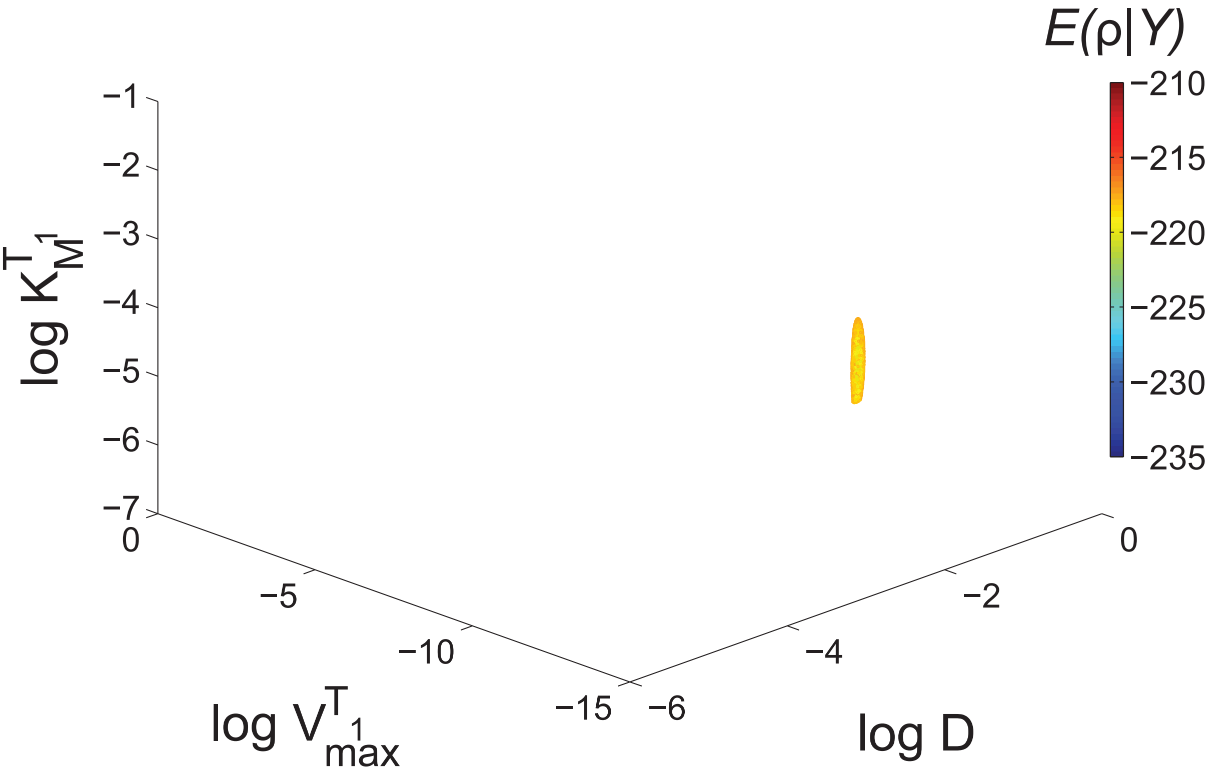

Figure 5: **Parameter distributions of the viable space for the glutamine Michaelis-Menten model.** We sampled $n = 49,538$ uniformly distributed viable points ($E(\boldsymbol{\rho}|\mathcal{Y}) < E(\boldsymbol{\rho}|\mathcal{Y})_v = -217.63$), with $E_{\rm min} = -220.41$ and $\langle E(\boldsymbol{\rho}|Y)\rangle = -218.66$. Some parameters appear to extend wider in parameter space, specially $K_{\rm M}^{\rm T_1}$ while others show more restricted distributions, like D and specially $V_{\rm max}^{\rm T_1}$. Figure shows the parameter ranges explored, in log scale.

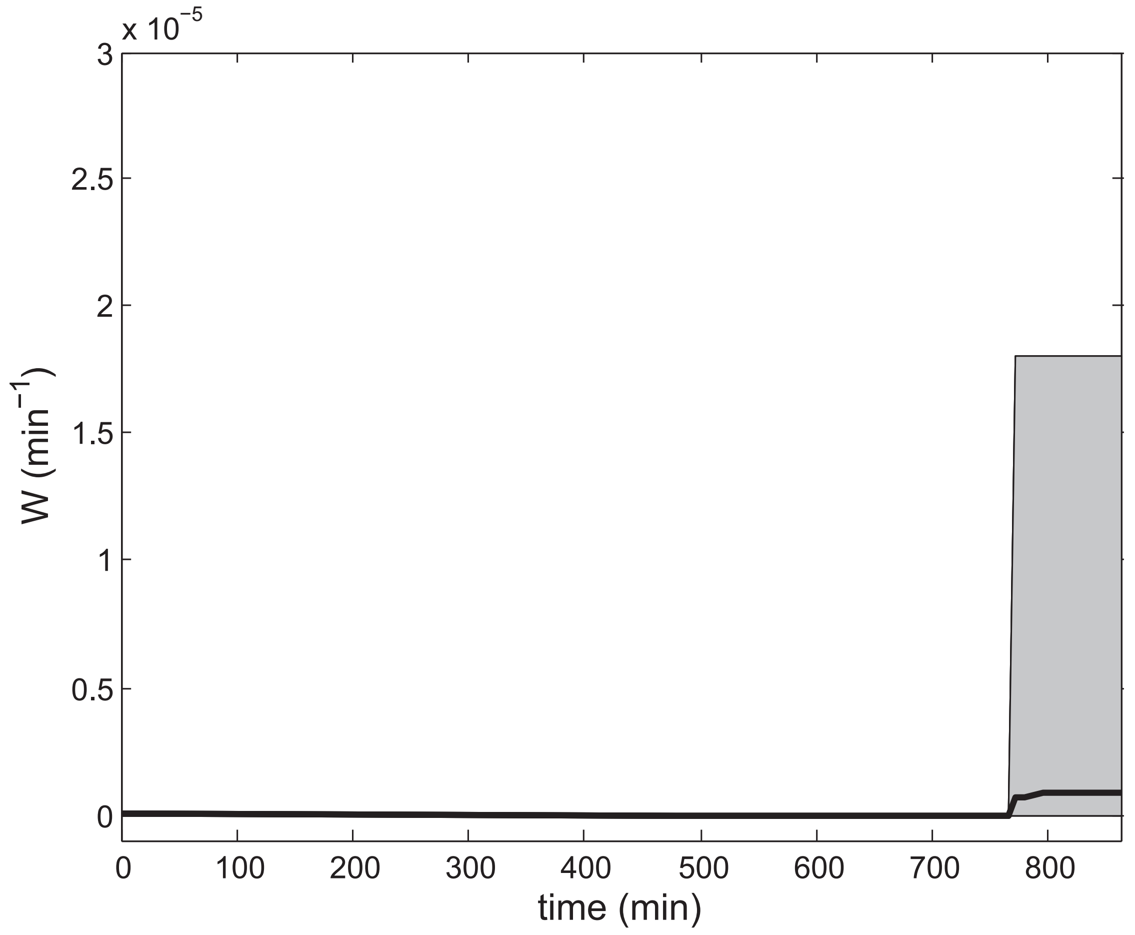

Figure 6: **Yeast glutamine transport missing term.** Prediction of state variable k_{A}, where the grey area is the 95% confidence interval for the prediction.

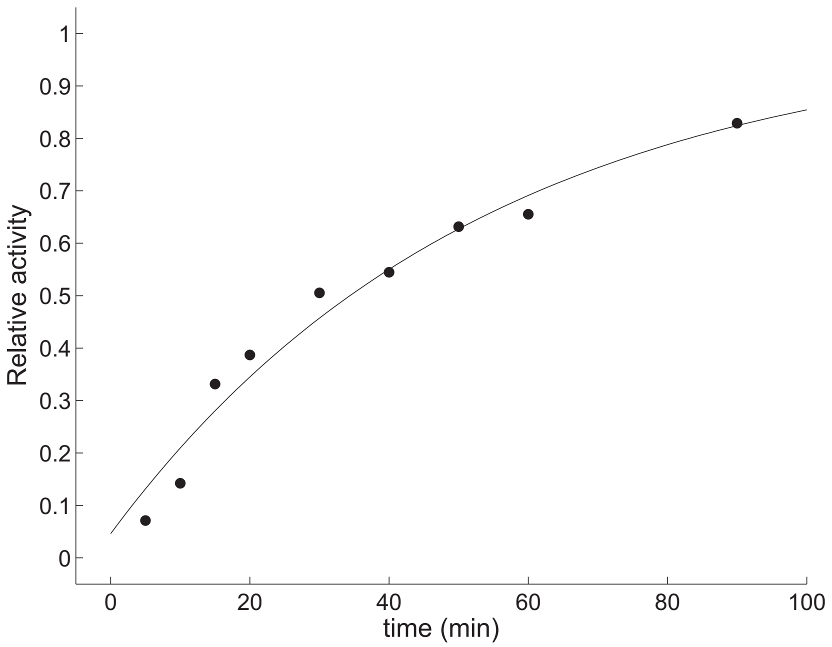

Figure 7: **Regulated activity.** Black dots represent the relative transport activity of Gap1 after amino acid depletion from the medium ($t = 0$) (Risinger *et al.*, 2006). The black line is the best fitting model $g(t) = 1 - (C_0\ e^{-k\ t})$ to the data.

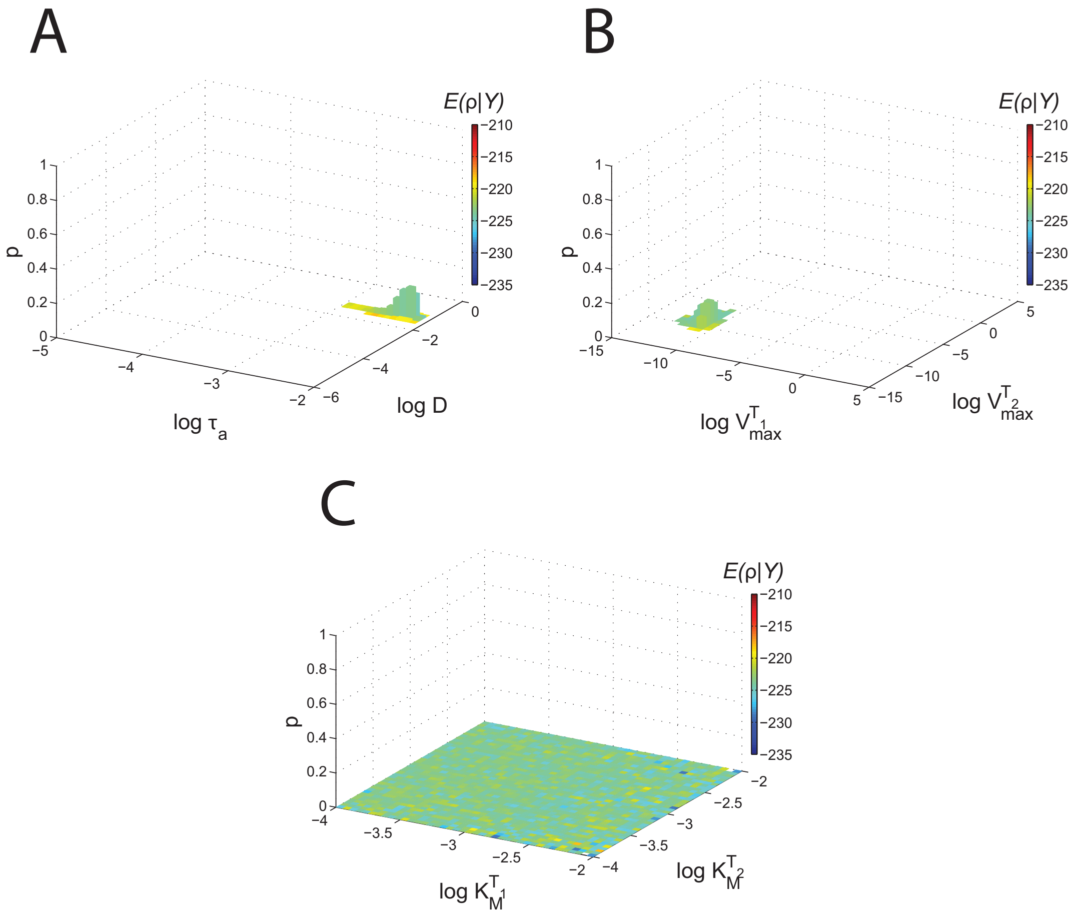

Figure 8: **Viable parameter space for the rMM model**. Projection of the viable parameter points to two of the parameters; **A**, τ_a and D, **B**, $V_{\mathrm{max}}^{\mathrm{T}_1}$ and $V_{\mathrm{max}}^{\mathrm{T}_2}$ and **C**, $K_{\mathrm{M}}^{\mathrm{T}_1}$ and $K_{\mathrm{M}}^{\mathrm{T}_2}$. The same color scale is used for $E(\boldsymbol{\rho}|Y)$ as in figure 4B in the main text. The best point sampled had a cost value of $E_{\mathrm{min}} = -234.26$ while the mean value of the parameter space is $\langle E(\boldsymbol{\rho}|Y)\rangle = -223.60$.

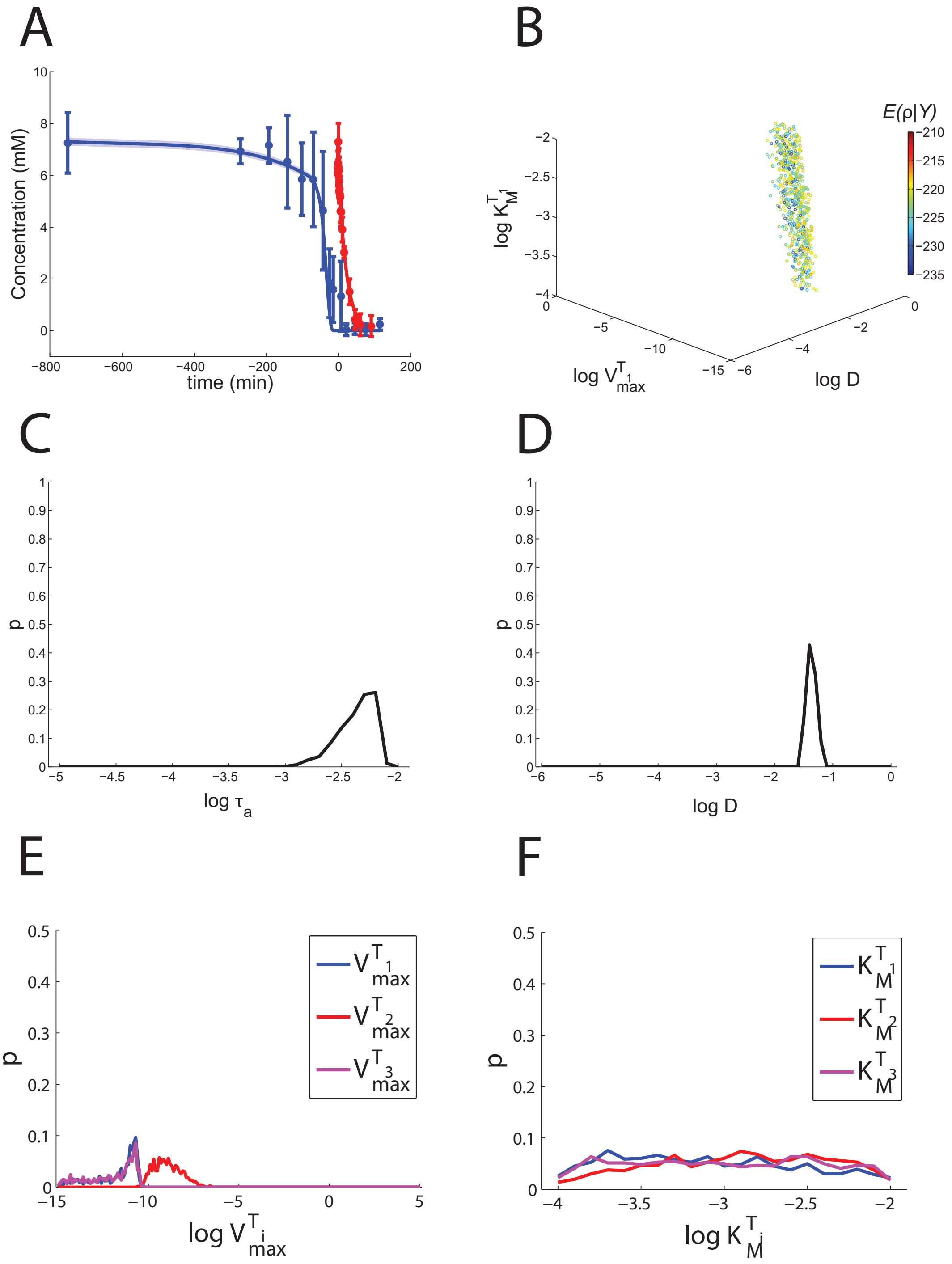

Figure 9: **e1rMM model results.** **A**, Model prediction and experimental data. Both observed dynamics, external and internal glutamine concentrations (blue and red dots respectively) are well explained by the e1rMM model. Lines represent the mean and shadowed regions the standard deviation of the trajectories built from the uniformly sampled viable space. **B**,The eight dimensional viable space (e1rMM model contains 8 structural parameters) is projected into three dimensions for visualization purposes. We sampled uniformly $n = 662$ points within the viable space.The cost value associated to each parameter point is mapped into a color scale. The best point sampled had a cost value of $E_{\min} = -233.48$ while the mean value of the parameter space is $\langle E(\boldsymbol{\rho}|Y)\rangle = -223.20$. **C-F**, Projection of the viable space for each parameter. The same color mapping as in **B** applies here to the mean $E(\boldsymbol{\rho}|Y)$ value of the parameter points laying at each bar of the histograms.

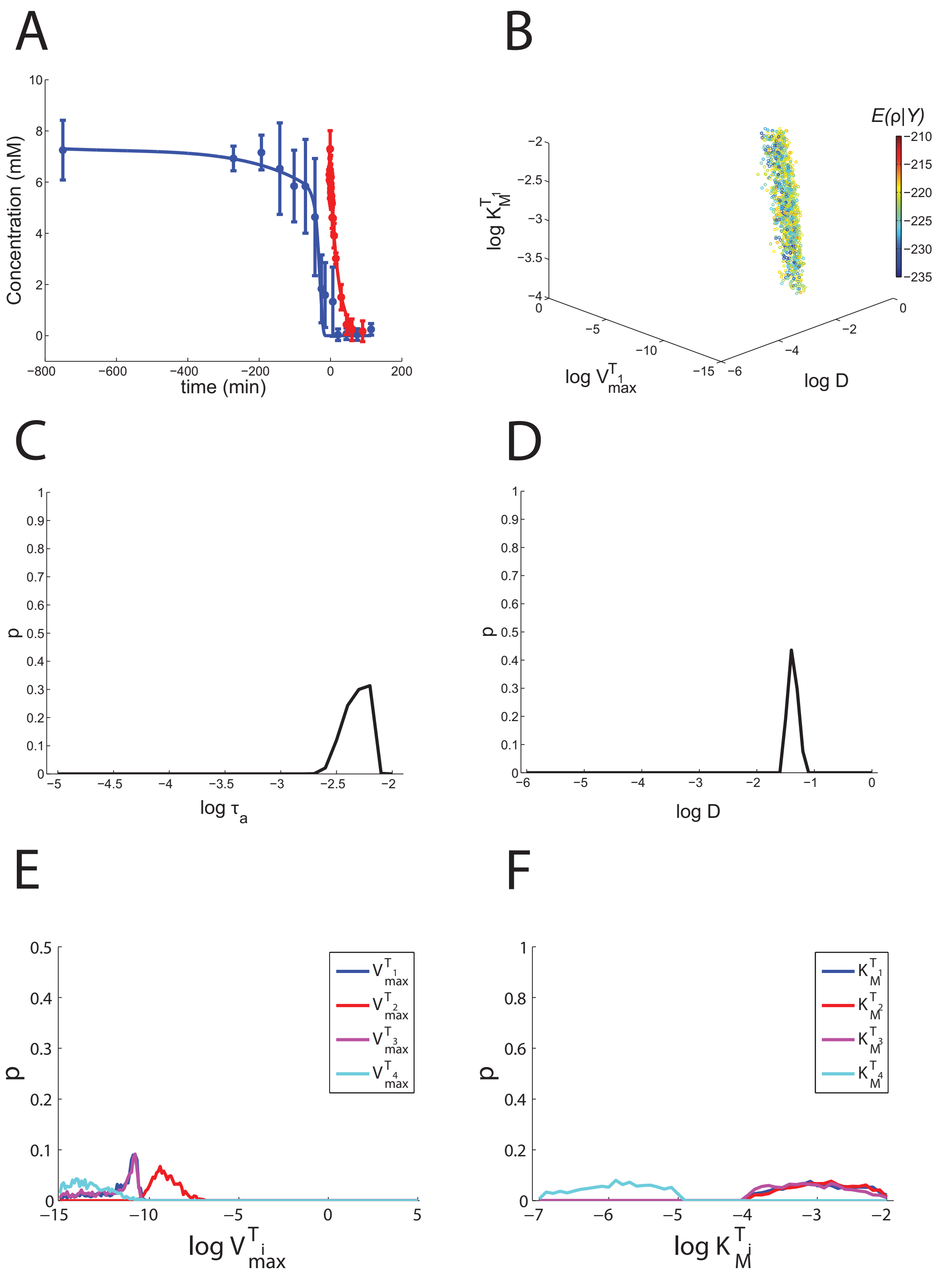

Figure 10: **e2rMM model results.** **A**, Model prediction and experimental data. Both observed dynamics, external and internal glutamine concentrations (blue and red dots respectively) are well explained by the e2rMM model. Lines represent the mean and shadowed regions the standard deviation of the trajectories built from the uniformly sampled viable space. **B**,The eight dimensional viable space (e2rMM model contains 8 structural parameters) is projected into three dimensions for visualization purposes. We sampled uniformly $n = 1,187$ points within the viable space. The cost value associated to each parameter point is mapped into a color scale. The best point sampled had a cost value of $E_{\min} = -233.81$ while the mean value of the parameter space is $\langle E(\boldsymbol{\rho}|Y)\rangle = -223.54$. **C-F**, Projection of the viable space for each parameter. The same color mapping as in **B** applies here to the mean $E(\boldsymbol{\rho}|Y)$ value of the parameter points located at each bar of the histograms.

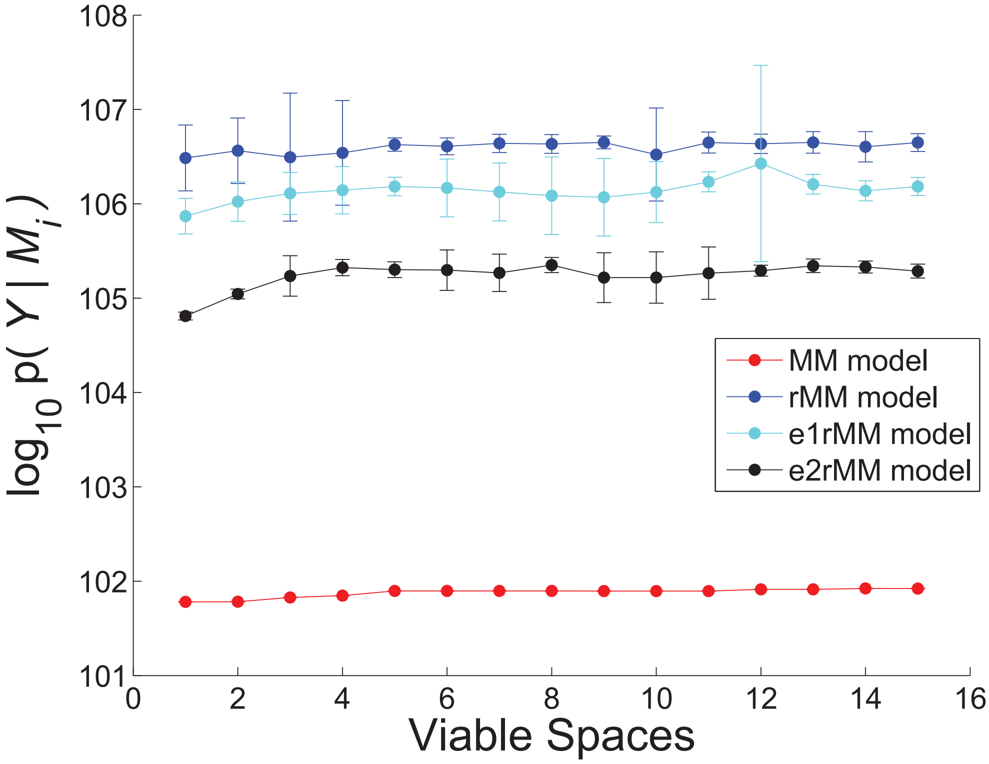

Figure 11: **Convergence of marginal likelihoods.** We did 15 independent explorations of the viable space for each model, and calculated the marginal likelihoods by integration over the parameter space (from which Bayes factors may be computed) from the union of n viable spaces. We repeated each calculations 20 times, which gives the standard deviations for each point. The marginal likelihoods of all models stabilize already after the union of a few viable spaces, which indicates that the viable spaces have been correctly identified.

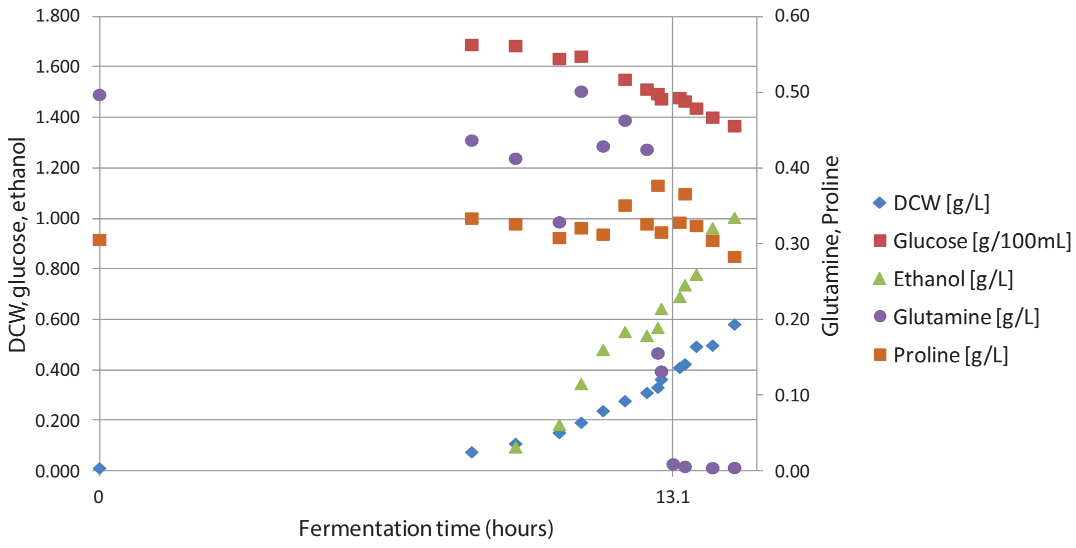

Figure 12: **Extracellular metabolite concentrations during a shift from glutamine to proline in batch culture.** The figure shows a representative experimental data from one of the three experimental replicates for the glutamine shift.
